# Immunization Gaps Among High-Risk Preterm Infants in Kazakhstan

**DOI:** 10.3390/vaccines14070638

**Published:** 2026-07-20

**Authors:** Balzhan N. Tatibekova, Daniel Yakubovich, Ademi K. Sagatbayeva, Aigul A. Ismailova

**Affiliations:** 1 Department of Epidemiology and Biostatistics, Astana Medical University, Astana 010000, Kazakhstan; 2 Center Katamneza (Pediatric Follow-Up Center), Astana 010000, Kazakhstan; 3 Department of Neonatology, Laniado Hospital, Ariel University, Netanya 42150, Israel

**Keywords:** preterm infants, delayed vaccination, immunization timeliness, measles outbreak, pertussis, SSPE

## Abstract

Background: Advances in neonatal intensive care in Kazakhstan have improved the survival of preterm infants, including those with very low birth weight (VLBW) and extremely low birth weight (ELBW). However, after hospital discharge, these medically vulnerable children may remain insufficiently protected against vaccine-preventable infections because of prolonged temporary medical exemptions, clinical caution, parental concerns, and fragmented coordination between specialized follow-up services and primary health care. Objective: This study aimed to assess vaccination coverage in a 10-year cohort of high-risk preterm infants and to identify clinical, parental, and organizational factors associated with delayed immunization. Methods: We conducted a retrospective cohort study of 331 high-risk preterm infants followed at a specialized pediatric center in Astana, Kazakhstan, between 2015 and 2024. Vaccination status at hospital discharge and at 24 months of chronological age, documented temporary medical exemptions, reasons for delayed or missed vaccination, and confirmed cases of pertussis and measles were analyzed. A supplementary cross-sectional survey of parents, physicians, and health care managers was conducted to further explore barriers to catch-up immunization. Results: At hospital discharge, 259 infants (78.2%) had not received the BCG or hepatitis B vaccine. By 24 months of age, 34.1% (n = 113) were fully vaccinated according to age, whereas 49.8% (n = 165) remained completely unvaccinated. During the observation period, 30 cases of measles and 17 cases of pertussis were recorded within the cohort, primarily during nationwide outbreaks. Among the 10 children with a history of measles before 12 months of age, 2 developed subacute sclerosing panencephalitis (SSPE), 1 developed profound hearing loss, and 1 required prolonged oxygen therapy following pneumonia. In addition, 2 children with periventricular leukomalacia (PVL) experienced clinical deterioration, while 1 infant with pertussis required hospitalization during the acute phase of illness. Conclusion: High-risk preterm infants in this cohort experienced substantial and persistent gaps in immunization after hospital discharge. These findings underscore the importance of regular reassessment of temporary medical exemptions, improved coordination between specialized follow-up services and primary health care, and strengthened catch-up immunization strategies for medically vulnerable children.

## 1. Introduction

Kazakhstan has achieved substantial reductions in neonatal mortality over the past decade, largely owing to advances in neonatal intensive care and perinatal services. As survival has improved, an increasing number of preterm infants, including those with very low birth weight (VLBW) and extremely low birth weight (ELBW), require long-term follow-up after hospital discharge [1,2].

In addition to developmental surveillance and management of chronic complications, these children require timely preventive care, including routine immunization [3]. International guidelines recommend vaccinating clinically stable preterm infants according to their chronological age, without correction for prematurity, unless specific contraindications are present [4,5,6,7]. Evidence also indicates that routine vaccines are generally safe and immunogenic in preterm infants [6,7].

Despite publicly funded immunization programs, delayed vaccination remains common among preterm infants [8,9]. Contributing factors include prolonged temporary medical exemptions, uncertainty among health-care providers, parental concerns, and inadequate coordination between specialized follow-up services and primary health care [10,11,12,13,14,15,16,17]. Consequently, vaccinations deferred during hospitalization may remain overdue after discharge.

This issue is particularly relevant in Kazakhstan, where improved survival of high-risk infants has coincided with recurrent outbreaks of measles and pertussis [18,19]. Specialized pediatric follow-up centers can help identify missed opportunities for vaccination; however, they are not always fully integrated into primary health-care vaccination pathways. As a result, children may undergo repeated specialist evaluations while remaining incompletely vaccinated.

The aim of this study was to assess vaccination status at 24 months of age among high-risk preterm infants, identify factors associated with incomplete vaccination, and describe the medical, parental, and organizational barriers to catch-up immunization.

## 2. Methods

### 2.1. Study Design and Setting

This retrospective cohort study was conducted among high-risk preterm infants born before 37 weeks of gestation with prematurity-related complications requiring long-term specialized follow-up. All children were followed at the Center for Coordinated Pediatric Follow-up Care in Astana, Kazakhstan, between 2015 and 2024.

The center is a specialized multidisciplinary medical facility that operates independently of the primary healthcare system. It provides specialized care for young children with complex medical conditions requiring long-term multidisciplinary management. Children are referred by their treating physicians or attend the center at their parents’ initiative. Because access to specialized follow-up programs remains limited in many regions of Kazakhstan, families from across the country attend the center for both in-person consultations and remote follow-up.

Medical care is delivered by a multidisciplinary team that includes neonatologists, neurologists, ophthalmologists, geneticists, cardiologists, endocrinologists, rehabilitation specialists, and other pediatric subspecialists according to each child’s clinical needs. Since 2023, the center has also provided day-hospital services.

Enrollment into the follow-up program was not standardized and depended on physician referral or parental initiative, clinical needs, and geographic accessibility. Consequently, children entered the program at different chronological ages, ranging from early infancy to later childhood.

The center does not administer routine childhood vaccines and is not integrated into the national immunization delivery system. In Kazakhstan, BCG and hepatitis B vaccines are routinely administered in maternity hospitals during the neonatal period, whereas subsequent childhood immunizations are provided free of charge through primary healthcare facilities according to the child’s place of registration and the National Immunization Schedule.

As part of the follow-up program, vaccination records were reviewed to assess immunization status, identify missed or delayed vaccinations, document temporary medical exemptions and parental refusals, provide vaccination counseling to families, and issue individualized catch-up immunization recommendations to primary healthcare providers.

For children with documented vaccine-preventable infections, additional retrospective data on the age at infection, year of infection, and major clinical outcomes were collected from medical records and information obtained during follow-up.

Because detailed assessment of clinical outcomes was not a predefined study objective, the availability and completeness of follow-up data varied among patients.

### 2.2. Study Population and Participant Selection

The study included all eligible preterm infants who were followed at the Center for Coordinated Pediatric Follow-up Care between January 2015 and December 2024. Eligible participants were children born before 37 completed weeks of gestation who had been referred to the center after discharge from perinatal centers or neonatal intensive care units. The study cohort therefore represents a consecutive clinical sample of preterm infants receiving specialized follow-up during the study period.

Children were included if vaccination status was documented at hospital discharge and/or during follow-up through 24 months of chronological age. Records were excluded if vaccination data were critically incomplete, vaccination status could not be verified, or follow-up information was insufficient for reliable outcome assessment.

A total of 604 children were initially screened for eligibility. During the initial screening, 118 cases were excluded, including 34 deaths and 84 children with incomplete clinical data. The remaining cohort comprised 486 children, of whom 342 were preterm and 144 were born at term. Because the study specifically aimed to evaluate immunization gaps among high-risk preterm infants, children born at term were excluded from the analytical cohort.

**Figure 1:**
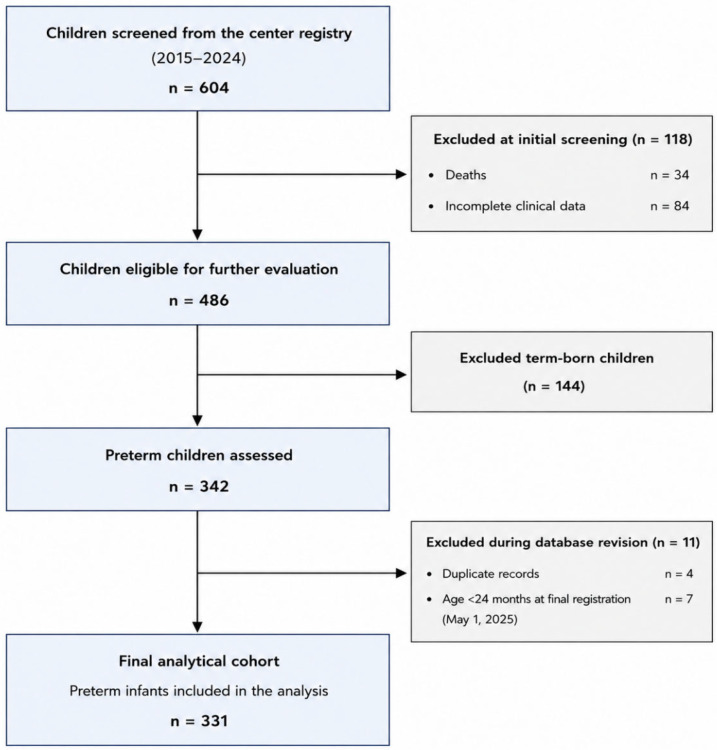
Flow diagram of participant selection and formation of the final analytical cohort.

During database verification, 11 additional cases were excluded, including four duplicate records and seven children with insufficient follow-up data to reliably assess vaccination completion according to the National Immunization Schedule. For the youngest children in the cohort, vaccination status was further verified during extended follow-up before completion of the final analysis. The final analytical cohort therefore consisted of 331 preterm infants. The participant selection process and formation of the final analytical cohort are shown in Figure 1.

The final cohort represented the full spectrum of prematurity, including extremely preterm, very preterm, and moderate-to-late preterm infants, and was not limited to the highest-risk or extremely low birth weight subgroups.

### 2.3. Vaccination Assessment and Data Collection

Information on vaccination status was obtained from neonatal discharge summaries, vaccination cards, outpatient and electronic medical records, specialist consultation reports, and documentation provided by primary healthcare facilities and families.

In Kazakhstan, BCG and hepatitis B vaccines are routinely administered in maternity hospitals during the neonatal period, whereas subsequent childhood immunizations are provided through primary healthcare facilities according to the National Immunization Schedule.

Vaccination status was evaluated at two predefined time points: hospital discharge and 24 months of chronological age. The assessment included receipt of BCG, hepatitis B, diphtheria–tetanus–pertussis-containing, poliovirus, Haemophilus influenzae type b (Hib), pneumococcal conjugate, and measles–mumps–rubella (MMR) vaccines.

For each child, information on vaccine administration, delayed vaccination, temporary medical exemptions, parental refusal, and catch-up immunization was recorded. When vaccination data were incomplete, additional information was obtained through follow-up visits, telephone interviews, and review of supplementary medical records provided by families.

Documented reasons for temporary medical exemptions and delayed vaccination were extracted from medical records and analyzed descriptively. Particular attention was given to vaccination status at 24 months, which was considered the primary time point for assessing completion of routine childhood immunization.

Full vaccination was defined according to the National Immunization Schedule of Kazakhstan applicable during the study period. Although minor changes in vaccine formulations occurred over time, the age-specific schedule and core vaccination components remained unchanged and did not affect the definition of vaccination completion.

Children were considered fully vaccinated if they had received all routine age-appropriate vaccine doses scheduled up to 24 months, regardless of whether individual doses had been administered later than recommended. Children with one or more missed scheduled vaccinations by 24 months were classified as partially vaccinated or unvaccinated.

To improve the accuracy of outcome assessment, reported cases of measles and pertussis were verified using outpatient medical records, hospital discharge summaries, available laboratory results, and information obtained during follow-up consultations.

### 2.4. Supplementary Analyses

To provide additional insight into factors associated with delayed and incomplete vaccination among preterm infants, supplementary analyses were performed alongside the retrospective cohort study.

A descriptive cross-sectional survey was conducted in April 2025 during European Immunization Week among parents of children followed at the center (n = 153), primary healthcare physicians and pediatricians involved in the care of high-risk children (n = 98), and healthcare administrators (n = 43).

The surveys were designed to explore perceived medical, parental, organizational, and healthcare system barriers to catch-up immunization. The parental questionnaire was developed as a multidimensional descriptive instrument based on the conceptual framework of vaccination barriers proposed by Cooper et al. [20]. Internal consistency and structural validity were evaluated using Cronbach’s alpha, the Kaiser–Meyer–Olkin measure, and Bartlett’s test of sphericity.

The surveys were not intended to retrospectively reconstruct parental or physician attitudes throughout the entire cohort observation period but rather to characterize contemporary perceptions and organizational barriers at the time of the study.

These supplementary analyses were undertaken because Kazakhstan does not have a dedicated national registry for systematically monitoring vaccination status, temporary medical exemptions, and catch-up immunization among high-risk preterm infants after discharge from neonatal care.

To provide national context for the cohort findings, immunization and epidemiological data were requested from the Ministry of Health of the Republic of Kazakhstan, the National Center for Public Health, and the Republican Center for Electronic Health. These data were used to compare the vaccination patterns observed in the study cohort with broader national trends and were not included in the primary retrospective cohort analysis.

### 2.5. Operational Definitions

A temporary medical exemption was defined as an officially documented temporary contraindication to vaccination recorded by a healthcare professional in the patient’s medical record.

Vaccination delay was defined as failure to receive a scheduled vaccine dose within the timeframe specified by the National Immunization Schedule of the Republic of Kazakhstan. Delay at 24 months was defined as failure to receive one or more vaccine doses scheduled during the first year of life by 24 months of chronological age.

A missed vaccination opportunity was defined as any healthcare encounter during which vaccination was not administered or a temporary medical exemption was not reassessed despite the absence of absolute contraindications.

Catch-up vaccination was defined as administration of previously missed vaccine doses following repeat clinical assessment.

### 2.6. Study Outcomes

The primary study outcome was vaccination status at 24 months of chronological age.

Secondary outcomes included documented cases of measles and pertussis identified during follow-up. Cases were identified through review of outpatient medical records, hospital discharge summaries, specialist reports, and follow-up information obtained from families.

Cases were classified as laboratory-confirmed when supported by polymerase chain reaction (PCR), serological testing, or other laboratory documentation. In the absence of laboratory confirmation, cases were considered clinically confirmed if the diagnosis had been documented by the treating physician in the medical record.

The occurrence of measles and pertussis was analyzed according to vaccination status at 24 months of age.

### 2.7. Bias Control

Several measures were implemented to minimize potential sources of bias inherent to the retrospective cohort design.

Selection bias was reduced by consecutively including all eligible preterm infants who attended the Center for Coordinated Pediatric Follow-up Care during the study period according to predefined eligibility criteria.

Information bias was minimized by verifying vaccination status using multiple independent data sources, including neonatal discharge summaries, vaccination records, outpatient and electronic medical records, documentation from primary healthcare facilities, and Supplementary Information obtained from families during follow-up when required.

To improve data completeness, missing or inconsistent vaccination information was resolved through repeated review of medical records and additional communication with families whenever possible.

The supplementary survey and national contextual data were used exclusively to support interpretation of the cohort findings and were not included in the primary retrospective cohort analysis.

Missing data for baseline clinical variables were infrequent (<5%) and were handled using available-case analysis without imputation.

### 2.8. Statistical Analysis

Statistical analyses were performed using Python 3.11.0. Descriptive statistics were used to summarize the characteristics of the study cohort. Categorical variables are presented as frequencies and percentages.

To characterize the medical vulnerability of the cohort, the association between gestational age and birth weight was assessed using Spearman’s rank correlation coefficient.

The occurrence of measles and pertussis was compared between fully vaccinated children and those with incomplete vaccination using Fisher’s exact test.

Associations between major neonatal complications, including severe bronchopulmonary dysplasia, grade III–IV intraventricular hemorrhage, and periventricular leukomalacia, and incomplete vaccination by 24 months of chronological age were evaluated using univariate analysis followed by multivariable logistic regression.

These variables were selected a priori based on their clinical relevance and severity, as they represent major neonatal conditions most likely to influence physicians’ decisions regarding temporary postponement of vaccination in medically vulnerable preterm infants. To maintain model stability and minimize the risk of overfitting, the number of predictors included in the final multivariable model was intentionally limited. Because gestational age, birth weight, and major prematurity-related complications are clinically interrelated, correlations among candidate predictors were examined before model construction, and highly collinear variables were not entered simultaneously into the final model. Cases with critically incomplete covariate data had been excluded during participant selection.

Results are reported as odds ratios (ORs), adjusted odds ratios (aORs), and 95% confidence intervals (CIs). All statistical tests were two-sided, and a *p*-value < 0.05 was considered statistically significant.

Survey responses from parents, physicians, and healthcare administrators were analyzed descriptively and are presented as frequencies and percentages. Internal consistency of the parental questionnaire was assessed using Cronbach’s alpha, while structural validity was evaluated using the Kaiser–Meyer–Olkin measure and Bartlett’s test of sphericity.

Aggregated national data covering the period from 2014 to 2024 were analyzed descriptively and used exclusively to provide epidemiological and healthcare system context for interpretation of the cohort findings. These data were not included in the inferential statistical analyses.

### 2.9. Ethical Considerations

The study protocol was approved by the Local Bioethics Committee of Astana Medical University (Decision No. 12, dated 10 April 2025).

Name of the Ethics Committee: Local Bioethics Committee of Astana Medical University.

Approval Code: Decision No. 12.

Approval Date: 10 April 2025.

The retrospective chart review was conducted using anonymized data. Written or electronic informed consent was obtained for participation in the 2025 survey. The study was conducted in accordance with the principles of the Declaration of Helsinki.

## 3. Results

The final analysis included 331 preterm infants. Overall, 70% of children were born very or extremely preterm, confirming that the cohort represented a medically vulnerable population. A strong positive correlation was observed between gestational age and birth weight category (Spearman’s r = 0.82; *p* < 0.001), consistent with the high prevalence of marked prematurity and low birth weight in the study cohort (Supplementary Figure S1). Detailed clinical characteristics are presented in Table 1.

### 3.1. Cohort Characteristics

The timing of entry into the follow-up program varied substantially across the cohort. Overall, 237 children (71.6%) entered follow-up before 6 months of age, 62 (18.7%) between 6 and 12 months, 25 (7.6%) between 12 and 24 months, and 7 (2.1%) after 24 months of age. Among the 113 children who were fully vaccinated by 24 months, most (97, 85.8%) had entered follow-up before 6 months of age, 13 (11.5%) between 6 and 12 months, and 3 (2.7%) between 12 and 24 months. None of the children who entered follow-up after 24 months achieved full vaccination by 24 months.

Analysis by birth cohort revealed marked variation in full vaccination coverage over the study period (Supplementary Table S1). Vaccination coverage remained relatively stable during the COVID-19 pandemic (2020–2021), with no apparent disruption in immunization continuity within this cohort. In contrast, full vaccination coverage declined in later birth cohorts (2022–2024). This pattern may partly reflect expansion of the center’s referral network and the increasing referral of medically complex infants from multiple regions of Kazakhstan, many of whom entered specialized follow-up at older ages, potentially delaying initiation of routine immunization.

### 3.2. Vaccination Status and Vaccine-Preventable Infections

At discharge from the perinatal center, 259 children (78.2%) had received neither BCG nor hepatitis B vaccine. Both vaccines had been administered to 36 children (10.9%), BCG alone to 30 (9.1%), and hepatitis B vaccine alone to 6 (1.8%).

Vaccination status at discharge did not necessarily predict subsequent completion of the National Immunization Schedule. During follow-up, many children remained unvaccinated because of prolonged temporary medical exemptions or parental refusal. By 24 months of chronological age, only 113 children (34.1%) were fully vaccinated for age, whereas 53 (16.0%) were partially vaccinated and 165 (49.8%) had received no vaccine doses. Among children with incomplete vaccination, temporary medical exemptions were documented in 68 children (20.5% of the total cohort), whereas parental refusal or persistent non-consent was recorded in 150 children (45.3%).

**Figure 2:**
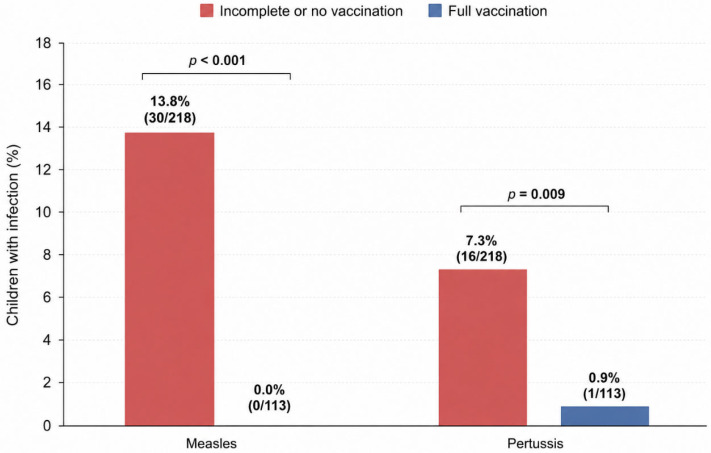
Vaccine-preventable infections among preterm infants according to vaccination status.

The occurrence of vaccine-preventable infections was analyzed according to age-appropriate vaccination status at the time of infection (Figure 2). Measles occurred in 30 of 218 children without complete age-appropriate vaccination and in none of the 113 fully vaccinated children (13.8% vs. 0.0%; Fisher’s exact test, *p* < 0.001). Pertussis occurred in 16 of 218 children without complete age-appropriate vaccination and in 1 of the 113 fully vaccinated children (7.3% vs. 0.9%; Fisher’s exact test, *p* = 0.009).

Among children with documented vaccine-preventable infections, temporal clustering was observed in relation to national outbreak periods. Of the 17 children with pertussis, one isolated case occurred in a child born in 2016 who developed pertussis in 2017, whereas the remaining cases were recorded during the national pertussis resurgence in 2023–2024. Among those followed longitudinally at our center, some children experienced repeated disease-related hospital readmissions.

Among the 30 children with measles, the most severe complications were documented during the 2019 outbreak and included two cases of subacute sclerosing panencephalitis (SSPE), one case of profound sensorineural hearing loss, and one case of post-infectious seizure exacerbation in a child with pre-existing periventricular leukomalacia. Most of the remaining measles cases occurred during the epidemic waves of 2023–2024, including one child with worsening seizure activity and one child with prolonged oxygen dependence following severe measles-associated pneumonia.

Detailed temporal and clinical characteristics of vaccine-preventable infections are summarized in Supplementary Table S2. Detailed clinical outcome data were available only for children who continued follow-up at our center, whereas infection histories in the remaining cases were obtained retrospectively from parental reports during follow-up visits. These findings are presented descriptively and should be interpreted with caution, as outcome assessment was not a predefined study objective and systematic follow-up data were not available for all infected children.

### 3.3. Factors Associated with Incomplete Vaccination

In univariate analysis, periventricular leukomalacia (PVL) was the only clinical factor significantly associated with incomplete or absent vaccination by 24 months of age (OR 2.48; 95% CI 1.34–4.61; *p* = 0.003). Neither severe bronchopulmonary dysplasia nor grade III–IV intraventricular hemorrhage demonstrated statistically significant associations with vaccination status (Supplementary Table S3).

In multivariable logistic regression analysis, periventricular leukomalacia (PVL) remained independently associated with incomplete or absent vaccination by 24 months of age (adjusted OR 2.47; 95% CI 1.32–4.63; *p* = 0.005), whereas severe bronchopulmonary dysplasia and grade III–IV intraventricular hemorrhage did not retain statistical significance after adjustment.

**Figure 3:**
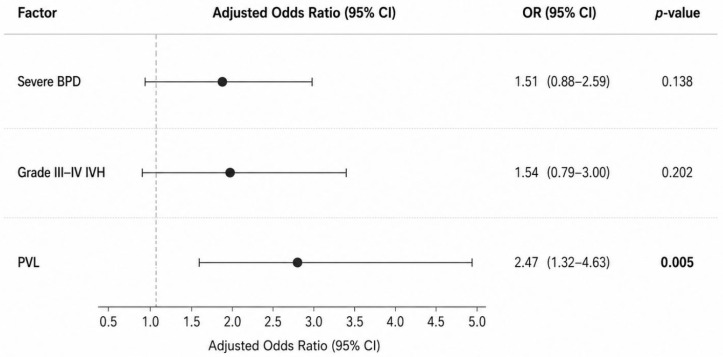
Clinical factors associated with incomplete vaccination by 24 months of age among preterm infants.

These findings are illustrated in the forest plot, where only the confidence interval for PVL remained entirely above the null value (OR = 1), whereas the confidence intervals for severe BPD and grade III–IV IVH crossed unity (Figure 3).

### 3.4. Contextual Survey Findings and National Epidemiological Data

To provide context for the observed immunization gaps, findings from the supplementary survey and national epidemiological data were analyzed descriptively.

Among physicians, the most frequently reported reasons for temporary medical exemptions were acute infectious diseases (88.7%), perinatal central nervous system pathology (60.2%), and anemia (48.0%). A history of seizures (69.4%) and cerebral palsy (61.2%) were also commonly cited as reasons for postponing pertussis-containing vaccines. Delays in BCG administration was primarily attributed to severe birth asphyxia and prematurity, whereas additional delays at the outpatient level resulted from the practice of waiting to assemble a sufficient number of children before opening a multidose vaccine vial. Only 18.7% of physicians reported being fully confident in vaccinating children with complex medical conditions, while 90.0% indicated that national vaccination recommendations should be revised.

Among parents, the leading reasons for vaccine refusal or delayed vaccination were concerns about vaccine safety (26.2%), the child’s underlying health condition (19.3%), and recommendations from healthcare professionals to postpone immunization (14.5%). Only 39.9% of parents reported receiving sufficient information from healthcare providers, whereas 45.8% indicated that additional counseling would facilitate decision-making regarding vaccination.

Among healthcare administrators, electronic vaccination registries and parental reminder systems were widely available. However, adapted vaccination clinic hours and outreach immunization services for children with limited mobility were available in only approximately half of the surveyed healthcare facilities. Detailed survey findings are presented in Supplementary Table S4.

National surveillance and vaccination monitoring data from Kazakhstan were additionally analyzed to provide epidemiological context for the cohort findings.

Analysis of DPT vaccination delays (Supplementary Figure S2) demonstrated that temporary medical contraindications consistently represented the leading reason for delayed immunization throughout the study period. Across most years, temporary contraindications accounted for the largest proportion of all documented causes, ranging from approximately 60% to nearly 80% of cases. Although the relative contribution of other factors, including vaccine shortages, parental refusal, and temporary transfers, varied over time, none demonstrated the same persistent predominance as temporary medical exemptions. Notably, even during periods when vaccine shortages substantially contributed to vaccination delays, temporary medical contraindications remained one of the leading causes of postponed DPT vaccination.

National neonatal statistics (Supplementary Figure S3) demonstrated an increase in the number of live-born infants in the low birth weight categories, accompanied by a steady decline in mortality within these groups. Over the study period, neonatal mortality in Kazakhstan decreased from 6.2 per 1000 live births in 2014 to approximately 4.0 per 1000 live births in 2024–2025, representing an overall reduction of nearly 35%.

This improvement was particularly evident among infants with very low birth weight (500–999 g) and low birth weight (1000–1499 g). Although the number of live-born infants in these vulnerable categories remained high or increased over time, the absolute number of deaths steadily declined. In the 500–999 g birth weight group, the number of live births ranged from 1231 to 2018, whereas deaths declined from 980 in 2014 to 750 in 2024. In the 1000–1499 g birth weight group, the number of live births ranged from 1352 to 2600, whereas deaths declined from 760 to 515 over the same period.

National infectious disease surveillance data (Supplementary Figure S4) demonstrated recurrent large-scale measles outbreaks in Kazakhstan throughout the study period, whereas pertussis incidence remained relatively low until a marked resurgence in recent years.

Measles incidence exhibited a pronounced cyclical pattern, with epidemic peaks in 2015, 2019, and 2023–2024. During the first nationwide outbreak in 2015, 2341 measles cases were reported, including 707 children aged <14 years. A second major epidemic occurred in 2019, when 13,326 cases were recorded, of which 9409 occurred among children younger than 14 years. The largest outbreak occurred in 2023, with 29,731 reported cases nationwide, including 23,536 children aged <14 years. Although incidence declined slightly in 2024, measles activity remained exceptionally high, with 28,147 reported cases, including 19,952 children younger than 14 years. By 2025, incidence had declined further to 4240 reported cases, of which 3679 occurred among children aged <14 years.

In contrast, pertussis incidence remained relatively stable throughout most of the observation period, with no major nationwide outbreaks before 2023. In 2023, 421 pertussis cases were reported, including 413 children younger than 14 years. In 2024, the number of reported cases increased substantially to 3110, including 3046 children younger than 14 years. By 2025, pertussis incidence had declined to 407 reported cases, of which 400 occurred among children aged <14 years.

Analysis of vaccination status among affected children identified two particularly vulnerable groups: children who had not yet reached the recommended age for vaccination and children with temporary medical exemptions. Among children with pertussis reported in 2023, 35 had not yet reached the recommended vaccination age, whereas 111 were unvaccinated because of temporary medical exemptions. In 2024, these numbers increased to 308 and 624, respectively.

A similar pattern was observed for measles. Among children with measles reported in 2023, 245 had not yet reached the recommended age for vaccination, whereas 441 were unvaccinated because of temporary medical exemptions. During the 2024 outbreak, these numbers increased markedly to 4801 and 1286, respectively.

## 4. Discussion

### 4.1. Main Findings

The findings of this study suggest that immunization gaps among preterm infants, including those born with very low and extremely low birth weight, may accumulate over time rather than occur as isolated events, potentially resulting in a cascade of missed vaccination opportunities. In our cohort, this process appeared to initiate as early as hospital discharge, when 78.2% of infants had not received routine neonatal vaccines. By 24 months of age, only 34.1% of children were fully vaccinated according to their chronological age, which might reflect the persistence and potential accumulation of missed immunization opportunities throughout early childhood.

Failure to receive neonatal BCG and hepatitis B vaccination could represent the first stage of this proposed cascade, a trend that appears consistent with observations in other cohorts of preterm infants and neonates requiring intensive care. Recent literature suggests that delays in neonatal vaccination may frequently be driven not only by clinical caution but also by potential organizational barriers within maternity hospitals and neonatal units [21,22,23,24].

According to our physician survey, even at the outpatient level, BCG vaccination was frequently postponed until a sufficient number of children were available to open a multidose vial. This operational constraint appeared to create a repeated barrier for children who had already missed vaccination at birth, which aligns conceptually with the phenomenon of missed opportunities for vaccination (MOV) described by the World Health Organization and discussed in other studies [25,26]. Crucially, the initiation of BCG and/or hepatitis B vaccination during the neonatal period did not inherently guarantee completion of the age-appropriate immunization schedule; many children subsequently experienced apparent delays in DTP-containing and other routine vaccines during outpatient follow-up.

Potential Drivers of Delayed Immunization

A second stage of the immunization delay cascade appears to emerge at the level of DTP-containing vaccines, where the major determinants may not necessarily be the neonatal complications themselves, but rather their subsequent clinical and organizational consequences. In our cohort, periventricular leukomalacia (PVL) remained the most consistent marker of incomplete vaccination, which might reflect perceived neurological vulnerability, prolonged temporary medical exemptions, and potentially delayed reassessment of vaccination eligibility in outpatient care.

This interpretation appears to be supported by our physician survey, in which neurological disorders, seizure history, and cerebral palsy were among the most frequently reported reasons for postponing vaccination. Although stable neurological conditions are generally not considered absolute contraindications to routine immunization, concerns related to neurological comorbidity, clinical uncertainty, and the frequent use of temporary exemptions may continue to contribute to further vaccination delays [27,28,29,30,31].

### 4.2. Clinical Context and Systemic Variations

The potential clinical relevance of these immunization gaps was highlighted by the occurrence of vaccine-preventable infections during follow-up. In our cohort, nearly all pertussis cases and all measles cases occurred among children with incomplete or absent vaccination, which may indicate persistent vulnerability during periods of active community transmission.

The most severe measles-related complications were observed among children infected during infancy, particularly before 12 months of age, and included two cases of subacute sclerosing panencephalitis (SSPE) and one case of severe sensorineural hearing loss. Notably, both children who later developed SSPE had been born preterm at 32 weeks of gestation. Although no causal relationship can be established, these observations may suggest that preterm infants who acquire measles early in life could represent a particularly vulnerable subgroup for severe long-term neurological complications. These findings are consistent with previous reports indicating that younger age at infection may be associated with an increased risk of severe neurological and respiratory outcomes in both measles and pertussis [31,32,33,34].

Although most children in our cohort entered specialized follow-up care within the first six months of life, and fully vaccinated children were more likely to have entered follow-up earlier, overall vaccination coverage by 24 months remained low. This suggests that early enrollment into specialized follow-up alone may not guarantee timely completion of the immunization schedule.

In contrast to national cohorts from the Netherlands, Israel, and China [35,36,37,38], where most preterm infants eventually caught up with the vaccination schedule, immunization gaps in our cohort appeared to persist substantially longer. This disparity might reflect a potential structural fragmentation of the healthcare system, wherein specialized follow-up centers can identify missed vaccinations but may not be integrated into state-funded immunization pathways and typically do not administer routine vaccines independently [39,40].

### 4.3. National Context and Behavioral Insights

Additional survey and national monitoring data provide further insight into the potential mechanisms underlying these delays. Physician survey findings showed that temporary medical exemptions, particularly in children with neurological conditions, remain one of the leading reported reasons for postponing vaccination, while only a small proportion of physicians reported full confidence in vaccinating high-risk children [41,42].

At the same time, parental survey findings indicated that delays were driven less by fixed anti-vaccination beliefs and more by potential concerns about the child’s health status, insufficient information, and recommendations from healthcare workers to postpone vaccination [43,44,45].

National surveillance data further showed that temporary medical exemptions remained the leading documented cause of delayed DTP vaccination in recent years, reaching their highest proportion in 2024, during a period marked by improved survival of preterm infants and recurrent measles and pertussis outbreaks.

Taken together, the national surveillance data and our institutional observations describe a similar epidemiological picture, characterized by frequent temporary medical exemptions, delayed vaccination, and the occurrence of vaccine-preventable infections among under-immunized children during periods of increased measles and pertussis incidence. Although these observations do not establish a direct causal relationship, they may reflect shared challenges in the immunization of medically vulnerable pediatric populations.

### 4.4. Clinical Implications

From a practical perspective, these findings point to a potential need to reconsider vaccination strategies for high-risk preterm infants at multiple levels [46,47,48].

Guideline Optimization: The changing clinical profile of surviving preterm infants, including a growing number of children with severe neurological and chronic complications, highlights the potential utility of updating national recommendations and improving physician training regarding the vaccination of this vulnerable population [49,50].

Neonatal Reassessment: At the neonatal stage, a more structured and earlier reassessment of temporary medical exemptions prior to discharge might help minimize missed BCG and hepatitis B vaccination opportunities [46,51]. For infants with very low and extremely low birth weight, further evaluation may be warranted to assess the feasibility and safety of earlier initiation of selected catch-up immunization stages, including DTP-containing vaccines, under clinical supervision within perinatal centers [52,53,54].

Primary Care Support: At the primary healthcare level, addressing organizational barriers, ensuring regular reassessment of contraindications, and strengthening provider competence in vaccinating children with complicated neonatal histories remain essential areas for consideration.

Systemic Integration: Given the decline in neonatal mortality and the increasing number of children surviving complicated neonatal periods, the integration of specialized follow-up programs into state immunization pathways appears to be a promising systemic strategy. This approach could potentially include the development of unified registries and integrated information systems capable of identifying children with special healthcare needs, thereby facilitating more individualized and adaptive immunization pathways [55,56,57].

## 5. Limitations

This study has several limitations. First, it was conducted in a single specialized follow-up center, which may limit the generalizability of the findings to other regions of Kazakhstan. Second, the retrospective design precludes causal inference regarding factors associated with vaccination status. Third, survey findings were based on self-reported responses and may be subject to reporting bias. Fourth, although the number of predictors in the multivariable model was intentionally restricted, the relatively limited number of fully vaccinated children may have affected the stability of the adjusted regression estimates. Fifth, the study did not include a comparator group of term-born children, which limits the ability to determine whether the observed vaccination delays are specific to preterm infants or reflect broader patterns of childhood immunization. Sixth, children who died or had incomplete follow-up data before final cohort selection were excluded from the analytical cohort, which may have introduced selection bias and potentially affected vaccination coverage estimates. Finally, detailed clinical outcomes of vaccine-preventable infections were available only for a subset of children who continued follow-up at our center, which limits the completeness of outcome assessment and the strength of clinical interpretation. Nevertheless, the study benefits from a long observation period, individual-level vaccination data, and the integration of clinical findings with contextual information from physician surveys and national epidemiological data.

## 6. Conclusions

This study identifies a persistent immunization gap among high-risk preterm infants within the studied cohort in Kazakhstan. Vaccination delays appeared to originate in the neonatal period and were subsequently sustained by a combination of clinical and organizational factors, including prolonged temporary medical exemptions.

These findings highlight a potential need for regular reassessment of medical exemptions, stronger coordination between specialized follow-up services and primary healthcare providers, and a wider implementation of catch-up immunization strategies for high-risk children. The data suggest that missed neonatal vaccination may represent an initial step in a prolonged, potential cascade of delayed immunization among medically vulnerable preterm infants.

## Figures and Tables

**Table 1:** Demographic and Clinical Characteristics of Preterm Infants by Vaccination Status.

Characteristic	Total Cohort (n = 331) n (%)	Fully Vaccinated n (%)	Partially Vaccinated n (%)	Unvaccinated n (%)
Sex				
Male	188 (56.8)	70 (37.2)	24 (12.8)	94 (50.0)
Female	143 (43.2)	43 (30.1)	29 (20.3)	71 (49.7)
Gestational Age				
Extremely preterm (<28 weeks)	123 (37.2)	43 (35.0)	22 (17.9)	58 (47.2)
Very preterm (28–31 + 6 weeks)	126 (38.1)	45 (35.7)	24 (19.0)	57 (45.2)
Moderate-to-late preterm (32–36 + 6 weeks)	82 (24.8)	25 (30.5)	7 (8.5)	50 (61.0)
Birth Weight				
ELBW (<1000 g)	142 (42.9)	48 (33.8)	24 (16.9)	70 (49.3)
VLBW (1000–1499 g)	92 (27.8)	33 (35.9)	18 (19.6)	41 (44.6)
LBW (1500–2499 g)	56 (16.9)	18 (32.1)	7 (12.5)	31 (55.4)
>=2500 g	41 (12.4)	14 (34.1)	4 (9.8)	23 (56.1)
Bronchopulmonary Dysplasia (BPD)				
Any BPD	133 (40.2)	40 (30.1)	27 (20.3)	66 (49.6)
Mild	20 (6.0)	7 (35.0)	5 (25.0)	8 (40.0)
Moderate	26 (7.9)	8 (30.8)	8 (30.8)	10 (38.5)
Severe	87 (26.3)	25 (28.7)	14 (16.1)	56 (64.4)
Intraventricular Hemorrhage (IVH)				
Any IVH	164 (49.5)	59 (36.0)	23 (14.0)	82 (50.0)
Grade I–II	108 (32.6)	45 (41.7)	15 (13.9)	48 (44.4)
Grade III–IV	56 (16.9)	14 (25.0)	8 (14.3)	34 (60.7)
White Matter Injury				
PVL	75 (22.7)	15 (20.0)	2 (2.7)	58 (77.3)

## Data Availability

The data presented in this study are not publicly available due to privacy, ethical, and institutional restrictions. The datasets include national health data provided by the Ministry of Health and clinical data from patients and parents at the study center, and therefore cannot be shared publicly.

## Supplementary Materials

The following supporting information can be downloaded at: https://www.mdpi.com/article/10.3390/vaccines14070638/s1. Supplementary Figure S1. Association between gestational age and birth weight categories in preterm infants. Supplementary Figure S2. Distribution of reported reasons for delayed DPT vaccination in Kazakhstan from 2014 to 2024. Supplementary Figure S3. Live-born and deceased infants by birth weight categories. Supplementary Figure S4. National trends of measles and pertussis incidence in Kazakhstan 2012–2025. Supplementary Table S1. Temporal trends in full vaccination coverage by birth year among high-risk preterm infants (2015–2024). Supplementary Table S2. Temporal and clinical characteristics of vaccine-preventable infections in preterm infants. Supplementary Table S3. Association between selected clinical risk factors and incomplete or no vaccination by 24 months of age in preterm infants. Supplementary Table S4. Comprehensive Structural Mapping Matrix, Variable Specifications, and Psychometric Properties of the Multi-Stakeholder Immunization Survey Across Physicians, Healthcare Managers, and Parents.

## Abbreviations

aOR, adjusted odds ratio; BCG, Bacillus Calmette–Guérin; BPD, bronchopulmonary dysplasia; CI, confidence interval; CNS, central nervous system; DTP, diphtheria–tetanus–pertussis; DTaP, diphtheria–tetanus–acellular pertussis; ELBW, extremely low birth weight; HBV, hepatitis B vaccine; Hib, Haemophilus influenzae type b; HRIF, high-risk infant follow-up; IVH, intraventricular hemorrhage; MMR, measles–mumps–rubella; MOV, missed opportunities for vaccination; NICU, neonatal intensive care unit; OR, odds ratio; PCR, polymerase chain reaction; PHC, primary healthcare; PVL, periventricular leukomalacia; SSPE, subacute sclerosing panencephalitis; VLBW, very low birth weight; VPDs, vaccine-preventable diseases; WHO, World Health Organization.
