# Supplementary material for: Immunization Gaps Among High-Risk Preterm Infants in Kazakhstan
Source: Vaccines (Basel). 2026 Jul 20;14(7):638. doi: 10.3390/vaccines14070638 (PMC13431342; doi:10.3390/vaccines14070638)
Supplement: Supplementary file 1 [file vaccines-14-00638-s001.zip › Supplementary Table S4. Comprehensive Structural Mapping Matrix, Variable Specifications, and Psychometric Properties of the Multi-Stakeholder Immunization Su.pdf]

I. Physicians Survey

Given the exploratory structural design of the developed 10-item instrument among the cohort of clinicians (($N = 98$)), data factorability and mathematical suitability were rigorously evaluated using the Kaiser-Meyer-Olkin (KMO) Measure of Sampling Adequacy alongside Bartlett's Test of Sphericity.

The KMO index was utilized to assess the degree of shared variance among the items and to determine if the sample size was sufficient to yield stable factor structures. The analysis produced an overall KMO value of (.616). According to Kaiser's established taxonomy for sampling adequacy, this score satisfies the standard methodological threshold of (>.60) and is classified as "mediocre" yet entirely admissible for exploratory analysis. This confirms that the patterns of correlation within the dataset contain an acceptable ratio of common variance to error variance, indicating that the sample size is adequate to proceed with structure-seeking statistical models.

Concurrently, Bartlett's Test of Sphericity served as the primary statistical test for data factorability. Bartlett's test evaluates the null hypothesis that the population correlation matrix is an identity matrix—meaning all variables are perfectly orthogonal (independent) and all off-diagonal correlation coefficients are exactly zero. For this dataset, Bartlett's test was highly significant, (chi^2(45) = 93.45), ($p < .001$). This definitive result rejects the null hypothesis of independence, proving that substantial, non-random systematic covariation exists across the items. This confirms that the data matrix is mathematically non-singular and possesses an underlying structural architecture highly suitable for multi-variable groupings or dimension-reduction techniques.

To evaluate internal consistency, the overall Cronbach's alpha coefficient was computed, yielding (alpha = .547), (95% CI [.400, .669]). While this value falls below the traditional (70) psychometric threshold, a granular review of the survey items reveals that this low value is a structural artifact of the question architecture rather than an inherent flaw in data quality.

Specifically, the instrument incorporates situational and behavioral frequency items (e.g., tracking the clinical rate of parental vaccine exemption requests over time) rather than subjective, latent psychological constructs. Because these items function as independent, environmental indicators—where an increase in one clinical frequency does not strictly dictate or depend on a linear increase in another—the psychometric assumptions of unidimensionality and reflective internal consistency are violated. Bartlett's highly significant result confirms that these multi-faceted indicators share powerful collective variance; however, because they capture independent clinical scenarios, they operate as a formative index rather than a reflective scale, rendering the traditional Cronbach's alpha an overly conservative and mismatched metric for this specific structure.

Factor	Meaning
Factor1	temporary medical exemptions + contraindications
Factor2	vaccination logistics + organizational barriers
Factor3	physician confidence + clinical decision making
Factor4	national recommendations + guideline relevance

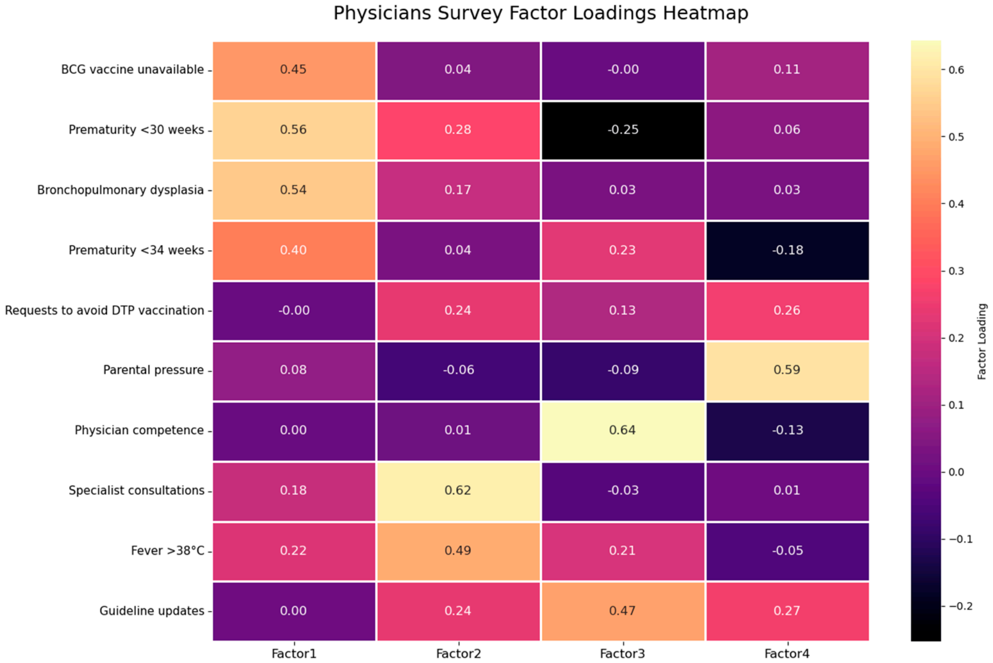

Factor	Eigenvalue	Decision
Factor1	2.15	Retained
Factor2	1.47	Retained
Factor3	1.25	Retained
Factor4	1.03	Retained
Factor5	0.89	Excluded
Factor6	0.77	Excluded
Factor7	0.72	Excluded
Factor8	0.64	Excluded
Factor9	0.60	Excluded
Factor10	0.48	Excluded

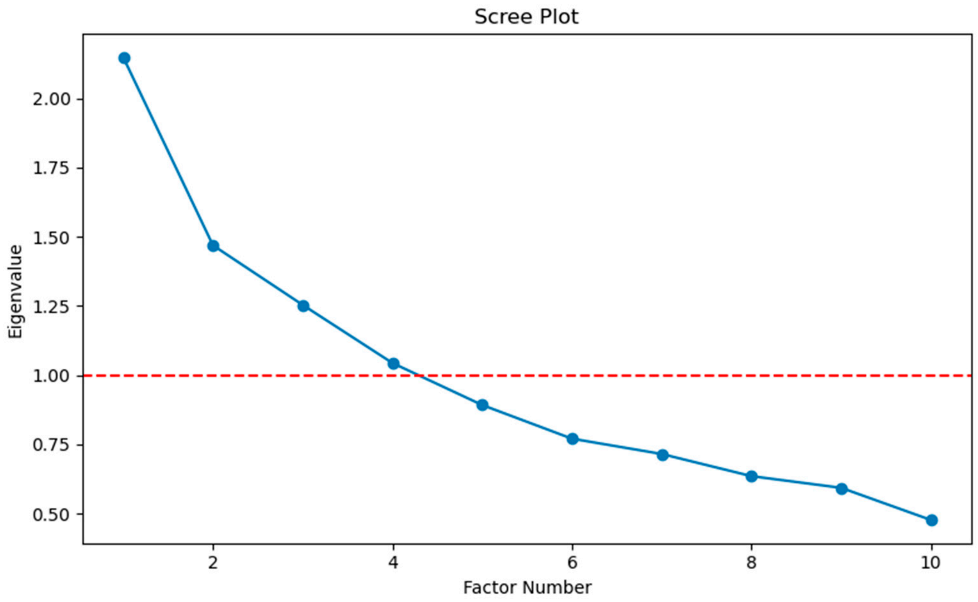

1.Factors interfering with BCG vaccination (if not performed in maternity hospital

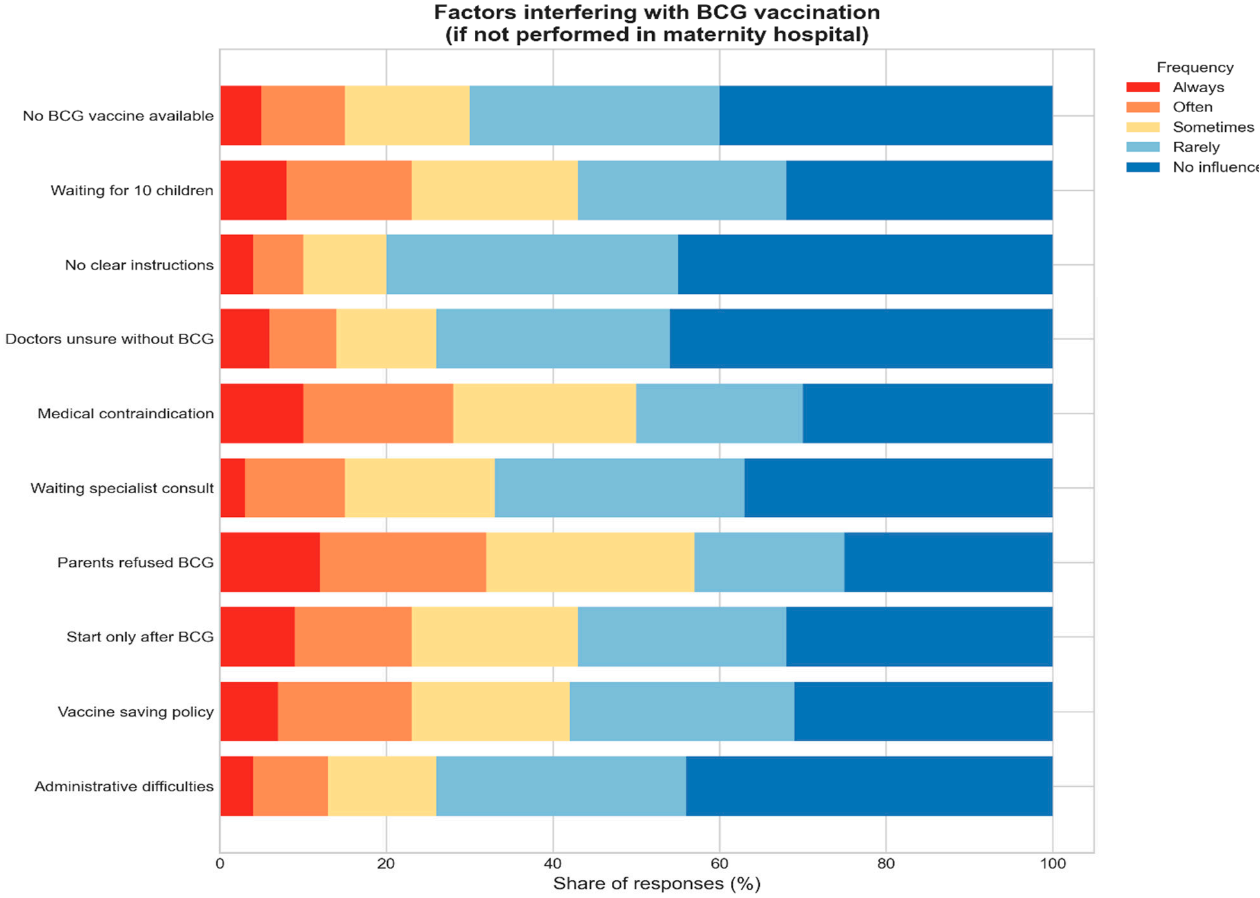

2. Grounds for medical exemption from pertussis vaccination (DTP)

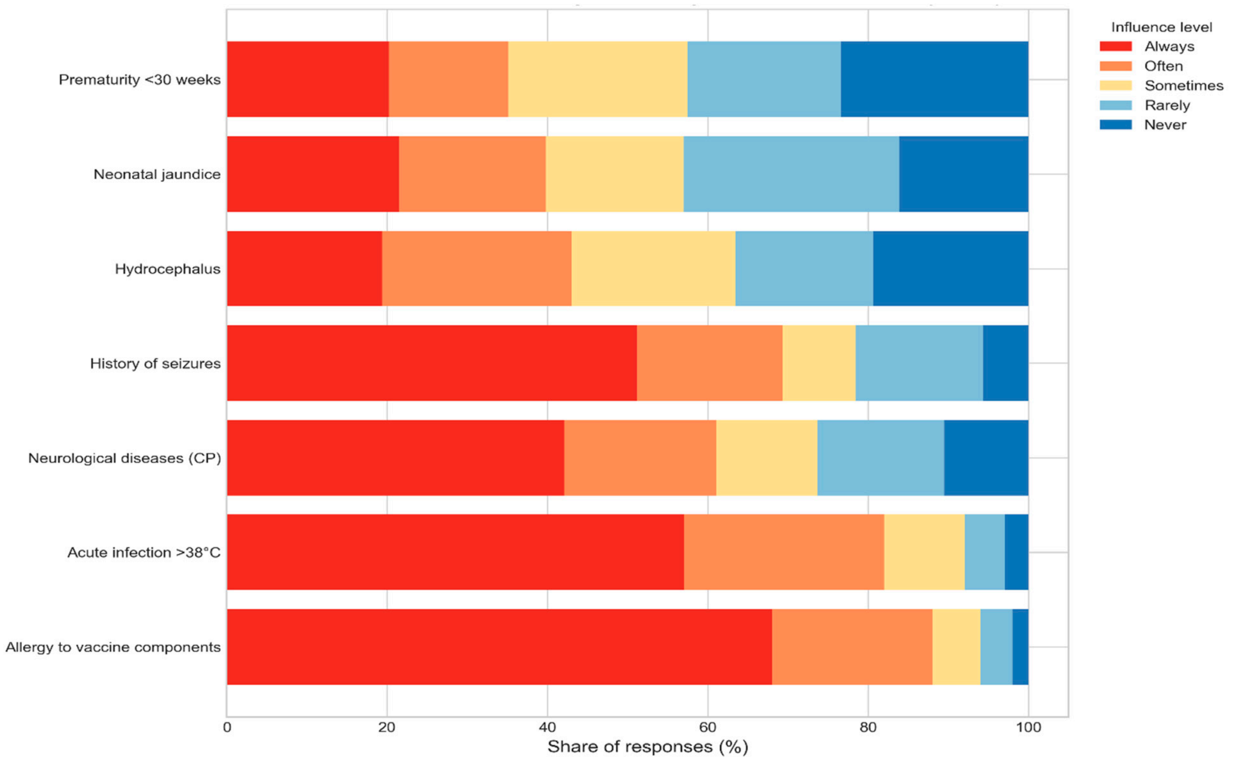

3. Grounds for medical exemption from MMR vaccination

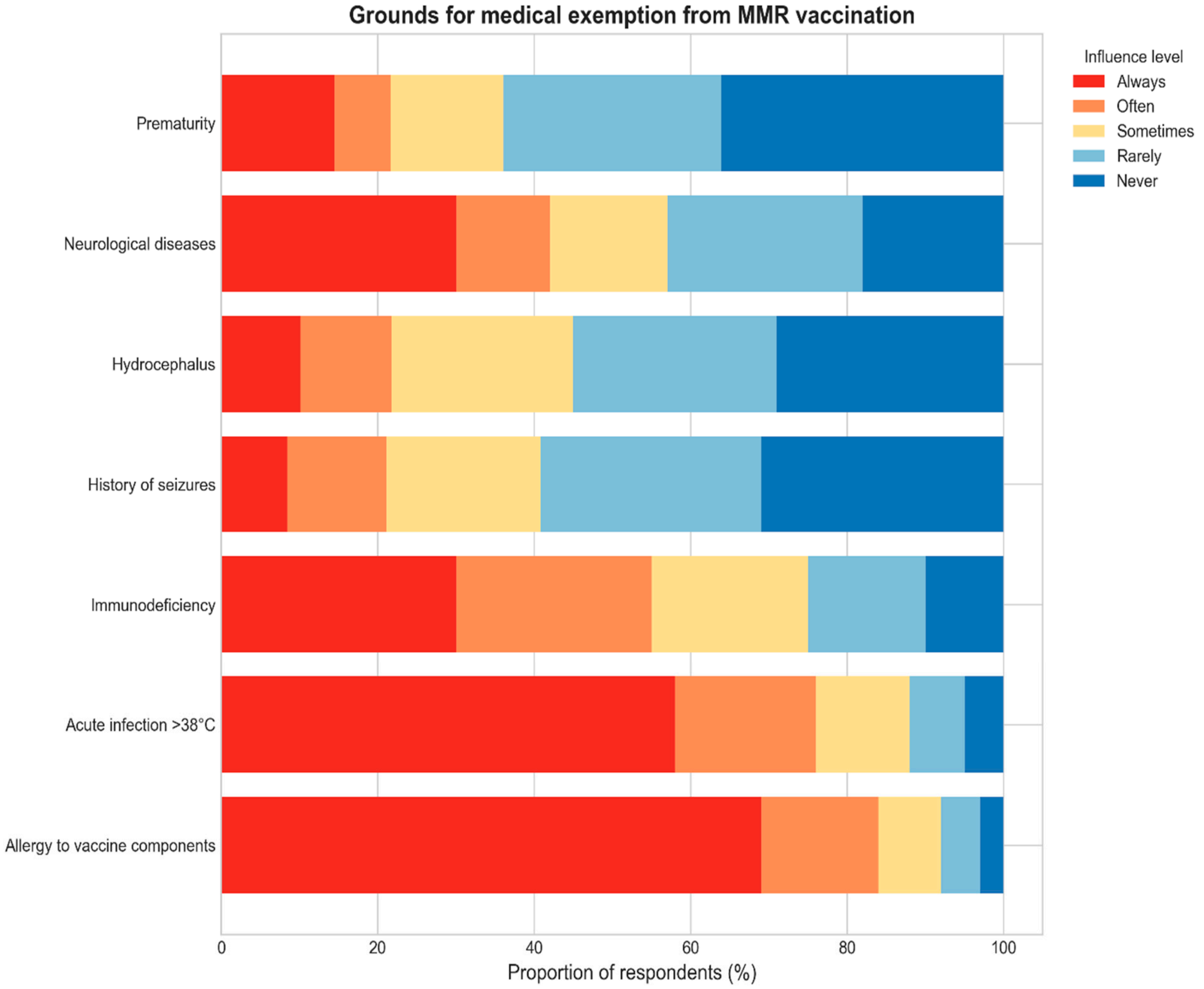

4. Conditions considered unacceptable for BCG vaccination in the maternity hospital

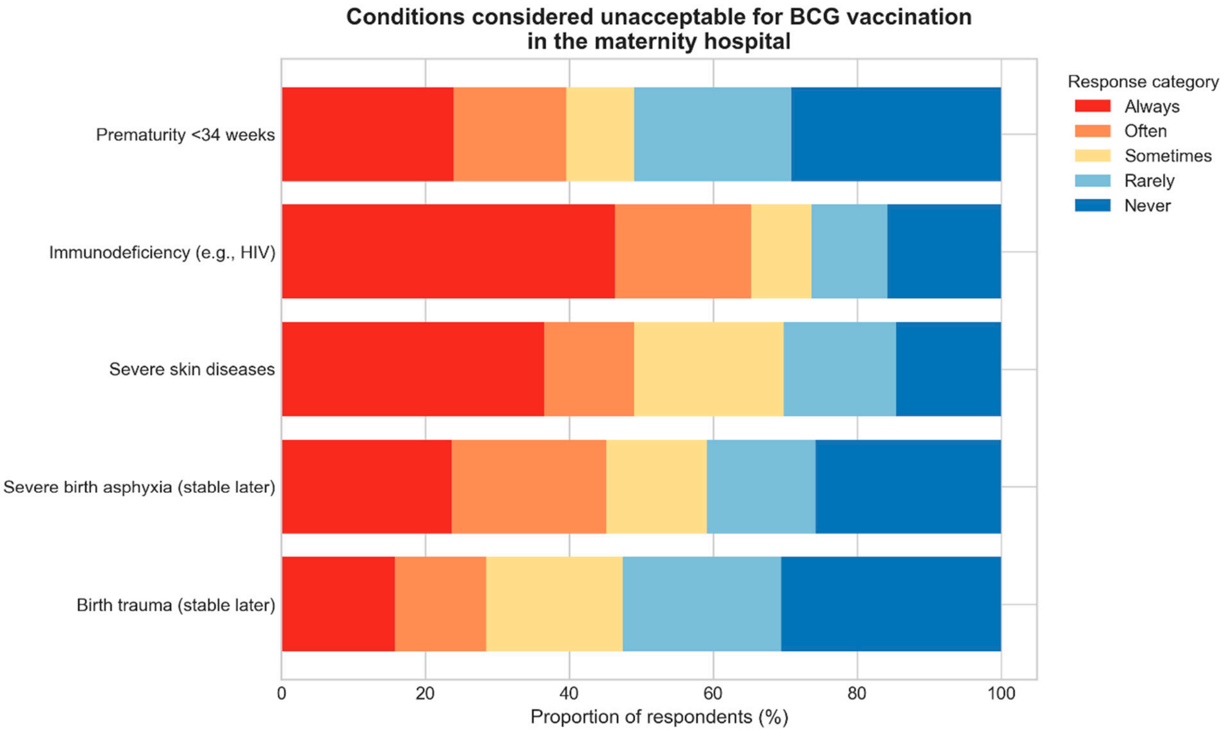

5. How often do you encounter parental requests for exemption from pertussis vaccination (DTP)?

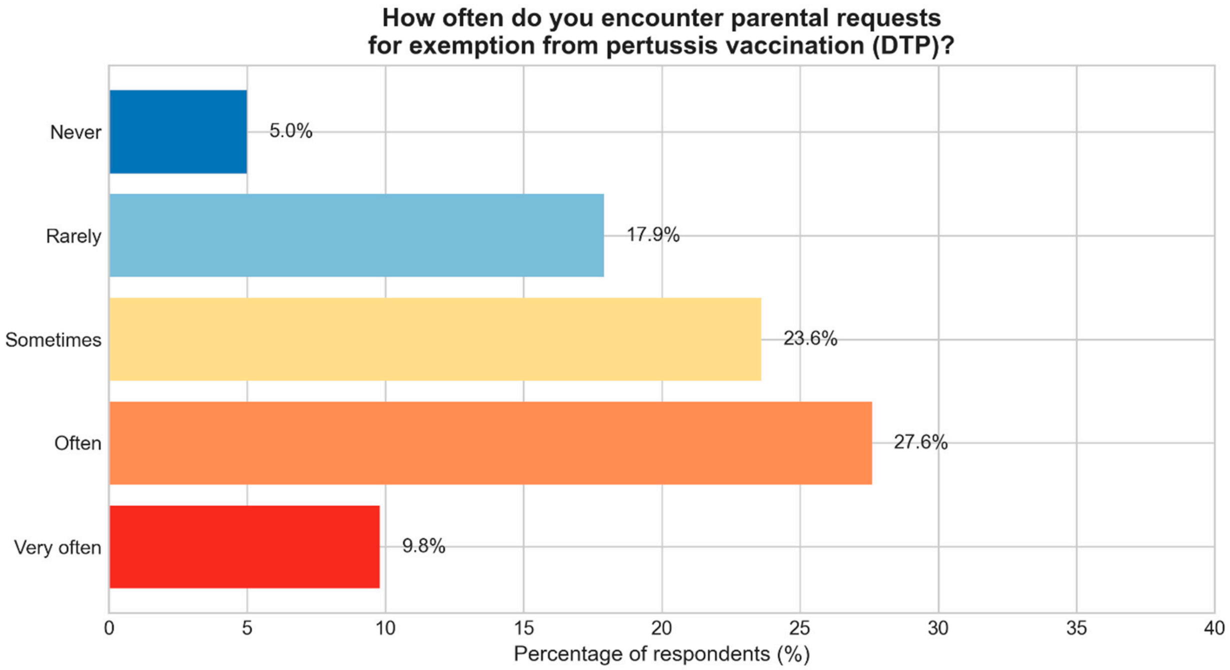

6. Factors influencing the decision to refuse vaccination

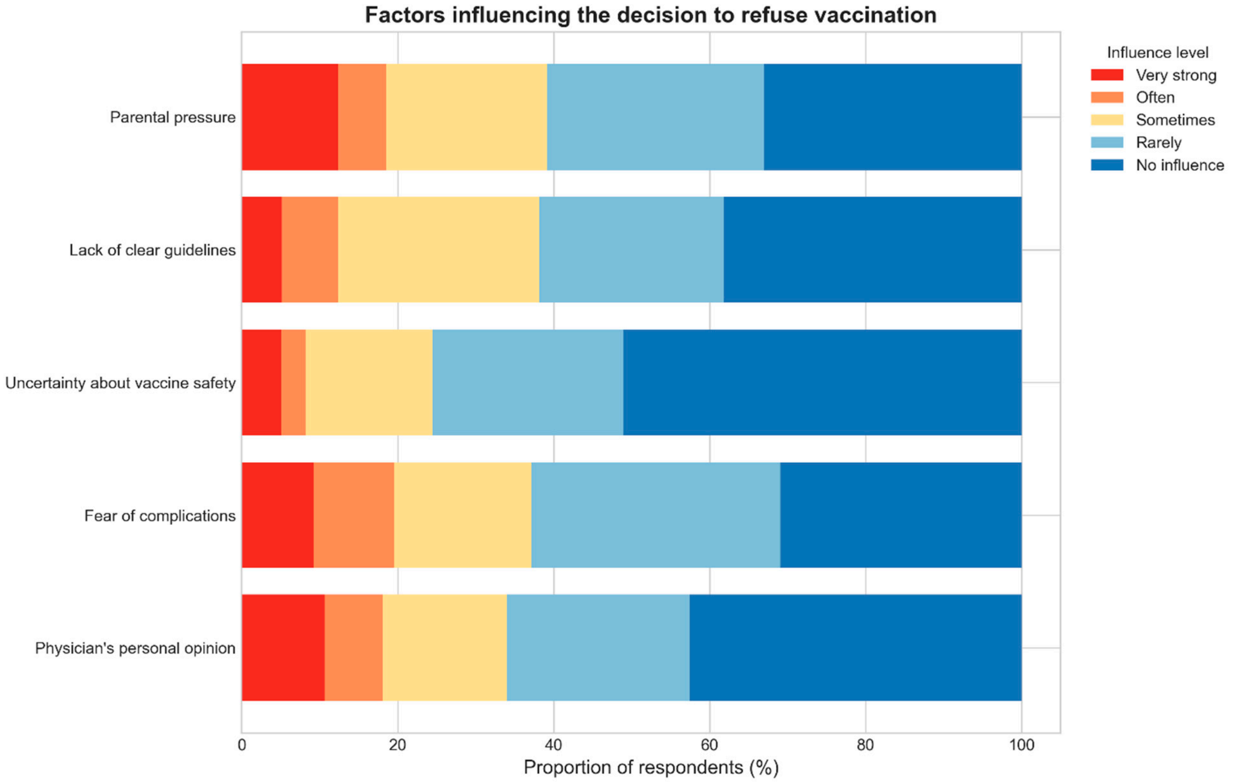

7. Confidence in professional competence when vaccinating children with chronic/neurological conditions

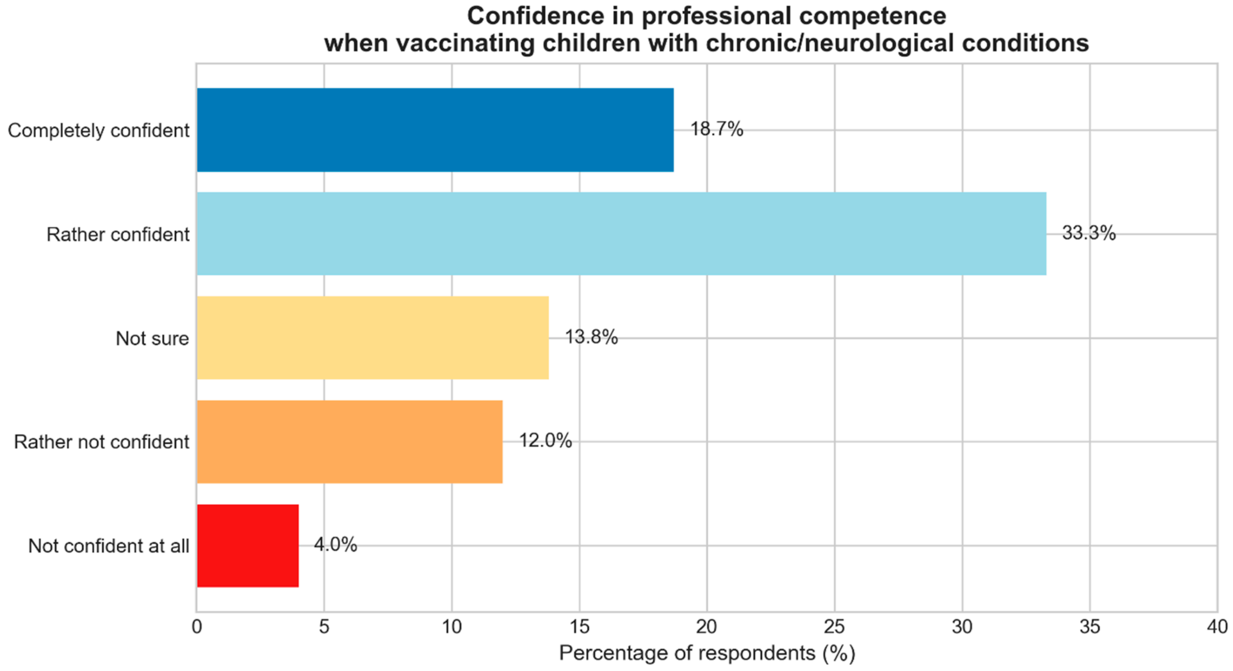

8. Frequency of consultations with specialists when making vaccination decisions for children with special needs

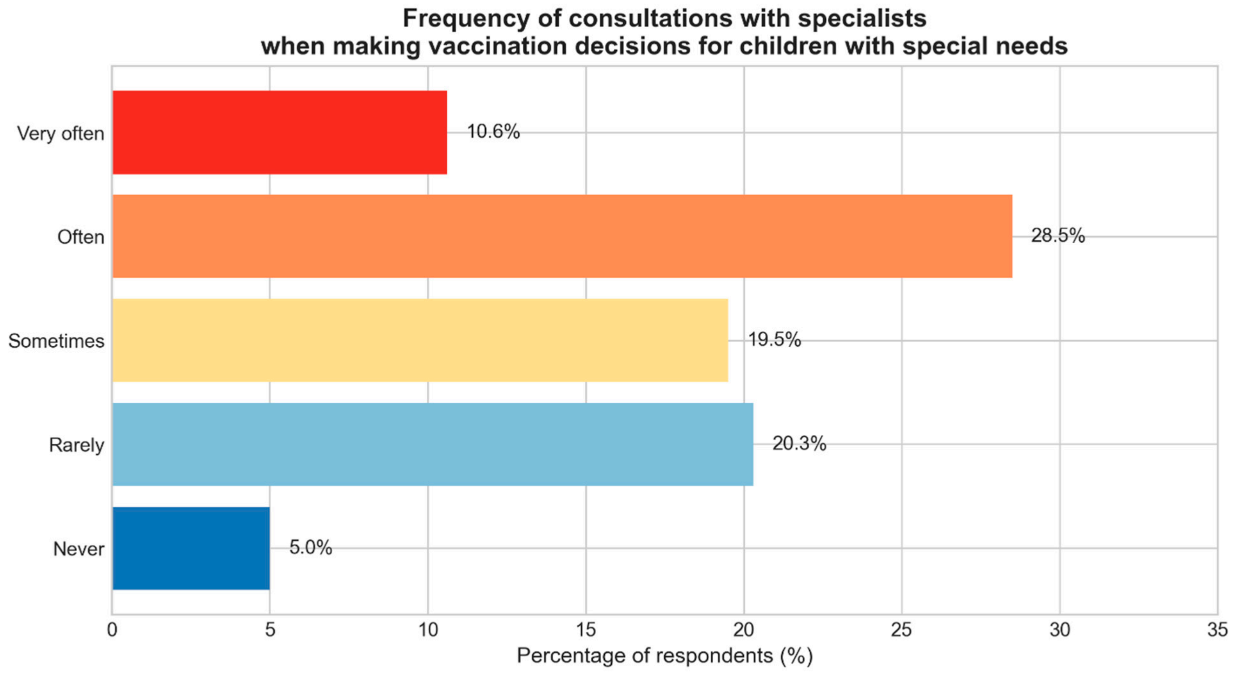

9. Conditions most frequently leading to temporary medical exemption from vaccination

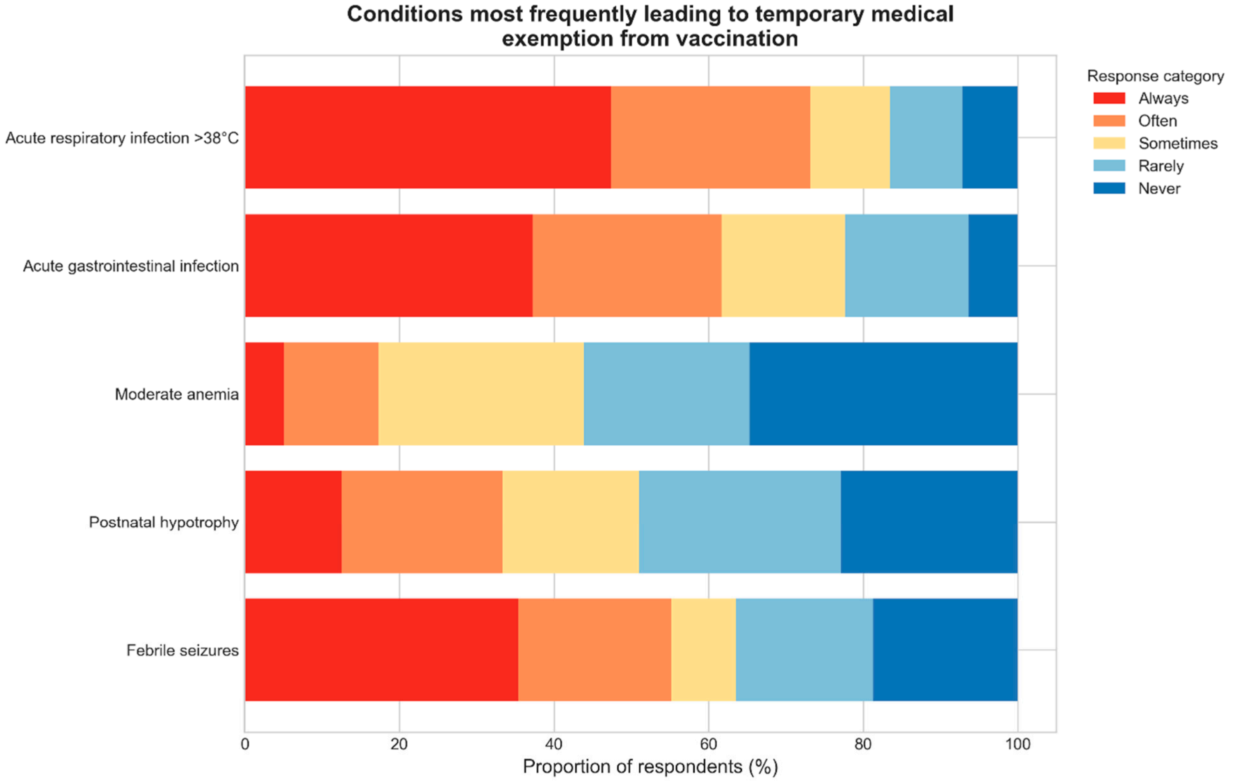

10. How do you assess the relevance of updating national vaccination recommendations for children with special medical needs?

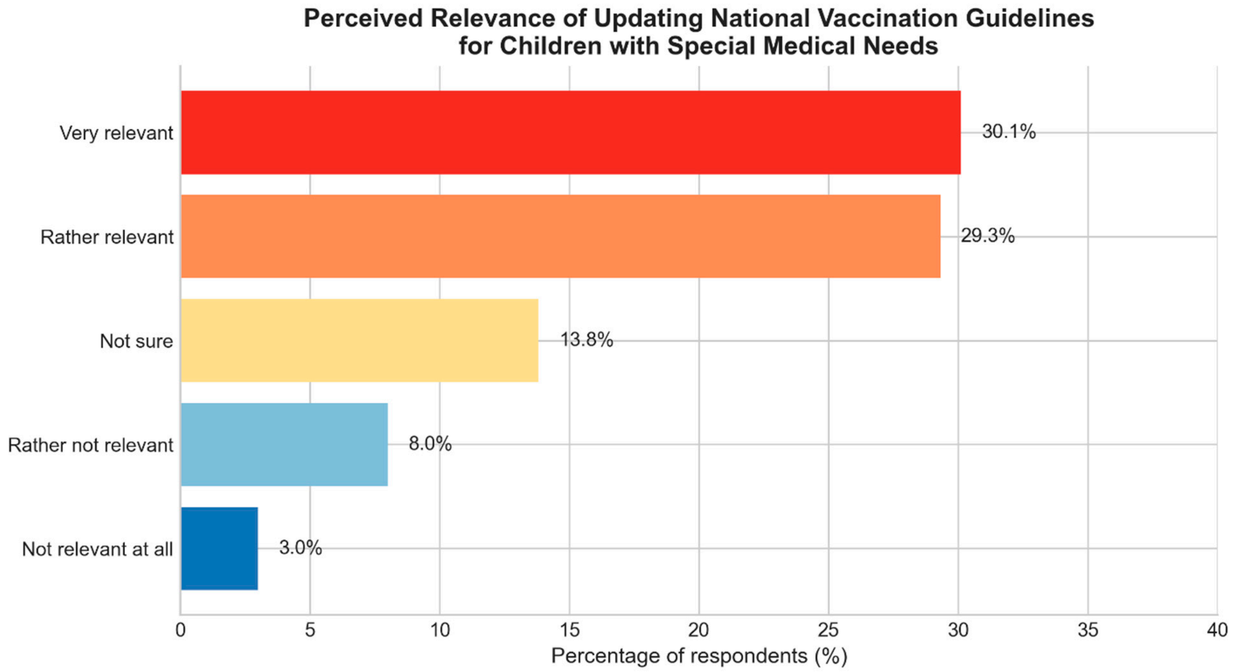

II.Parents Survey

1. METHODS SECTION

Questionnaire Design and Content Validity

To evaluate vaccination barriers, tracking accuracy, and parental perspectives within a specialized cohort of preterm and high-risk infants, a 14-item survey instrument (Items Q11–Q24) was developed. Because the instrument was designed to capture a broad spectrum of heterogeneous clinical, structural, and behavioral factors rather than a single psychometric trait, its content validity was established using a qualitative, framework-based approach.

The questionnaire items were systematically mapped against the evidence-based implementation and adaptation framework developed by Cooper et al. (2021) in their Cochrane Qualitative Evidence Synthesis. This matrix ensured that the survey items comprehensively address real-world vaccination barriers and remain consistent with international standards for exploring vaccine hesitancy.

Specifically, the survey elements were aligned with three core dimensions identified in the Cochrane synthesis:

Parental health beliefs and risk perceptions regarding specific infectious threats (such as pertussis and measles) and infant immune systems (Items Q16, Q17, Q18, and Q22);

Healthcare system accessibility, information quality, and structural care models, including parental attitudes toward the operational delineation of consultative roles between follow-up care centers and local primary care clinics (Items Q19, Q23, and Q24);

The quality of provider-patient communication, assessing specialized multi-disciplinary counseling by subspecialists (e.g., neurologists, immunologists) and the overall utility of medical information provided to families (Items Q20, Q21).

Baseline clinical and demographic characteristics, including child's age, gestational age at birth, and absolute immunization tracking status (Items Q11–Q15), were integrated to contextualize these behavioral and systemic domains within the specific medical vulnerabilities of the preterm population. The complete structural alignment and methodological rationale for each survey item are detailed in **Supplementary Table 1**.

2. DISCUSSION SECTION

Methodological Strengths of the Survey Instrument

A key methodological strength of this study lies in the structural framework of the parental questionnaire. Rather than utilizing a traditional, unidimensional psychological scale, the survey was built as an index of independent descriptive indicators, directly adapted from the Cochrane Qualitative Evidence Synthesis framework (*Cooper et al., 2021*).

As highlighted by Cooper and colleagues, vaccine hesitancy and immunization delays are driven by a complex, non-linear intersection of individual beliefs, institutional trust, and logistical healthcare delivery. By structuring our instrument around these internationally recognized Cochrane dimensions, we ensured that the questionnaire comprehensively captures the granular reality of barriers faced by parents of vulnerable, preterm infants.

Evaluating these diverse attributes as distinct, independent parameters—rather than forces trying to achieve artificial internal consistency or statistical aggregation—allows for a more precise, item-by-item analysis of systemic gaps. This approach directly aligns with the Cochrane recommendation for adapting immunization interventions to local institutional and parental realities, making the findings highly actionable for pediatric follow-up care systems.

THE COCHRANE CITATION (For your Reference List)

Link: Cooper S, Schmidt BM, Sambala EZ, et al. Factors that influence parents' and informal caregivers' views and practices regarding routine childhood vaccination: a qualitative evidence synthesis. Cochrane Database Syst Rev. 2021;10(10):CD013265. doi:10.1002/14651858.CD013265.pub2

11. Your child's age:

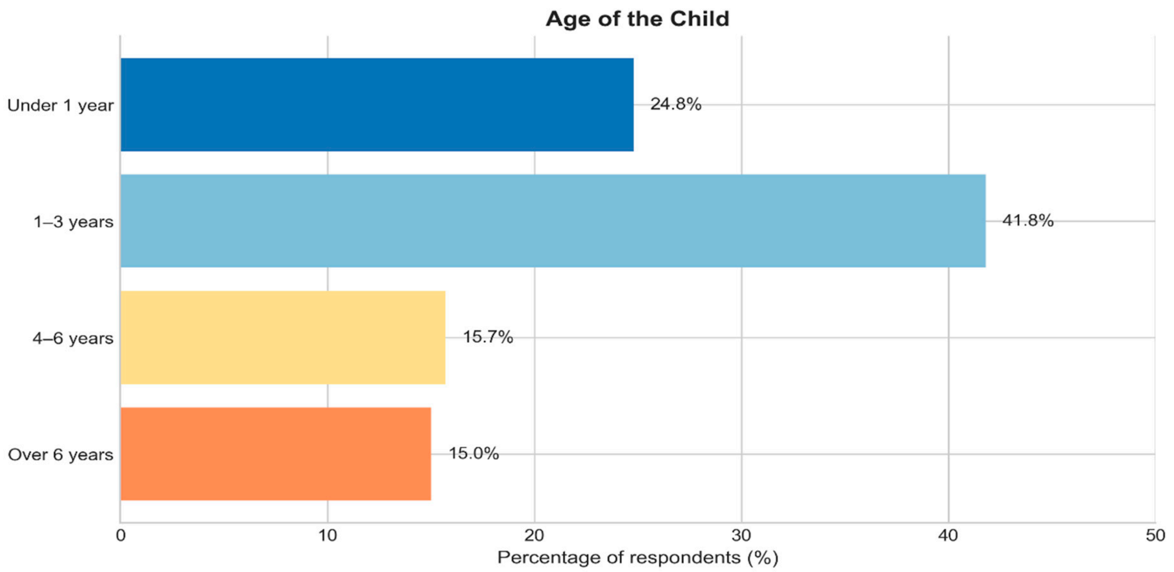

12. Gestational age at the birth of your child

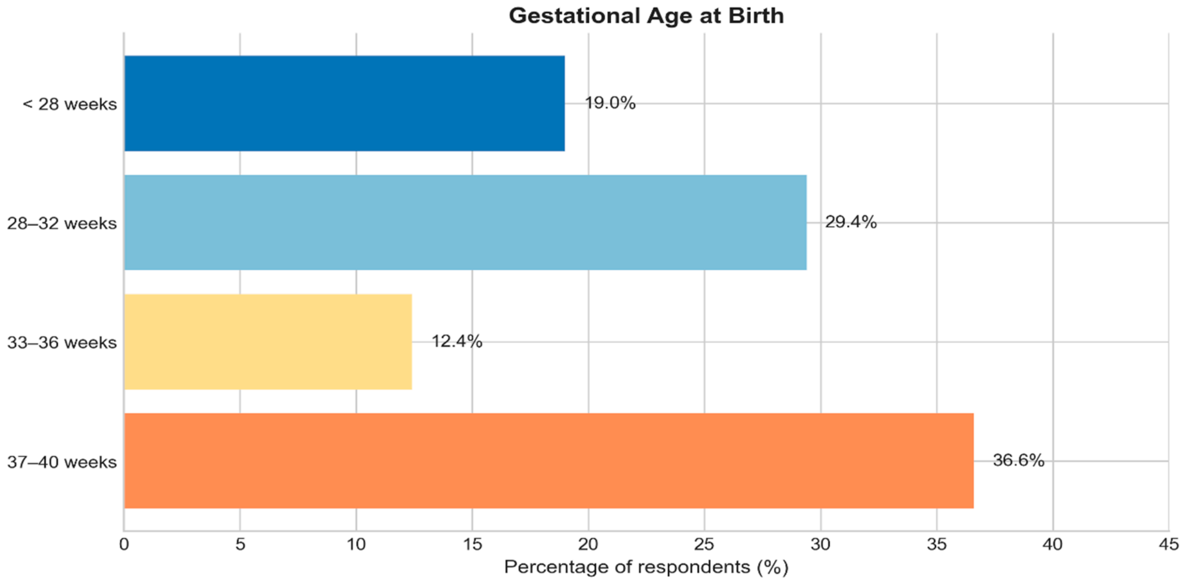

13. Has your child received all recommended vaccinations to date?

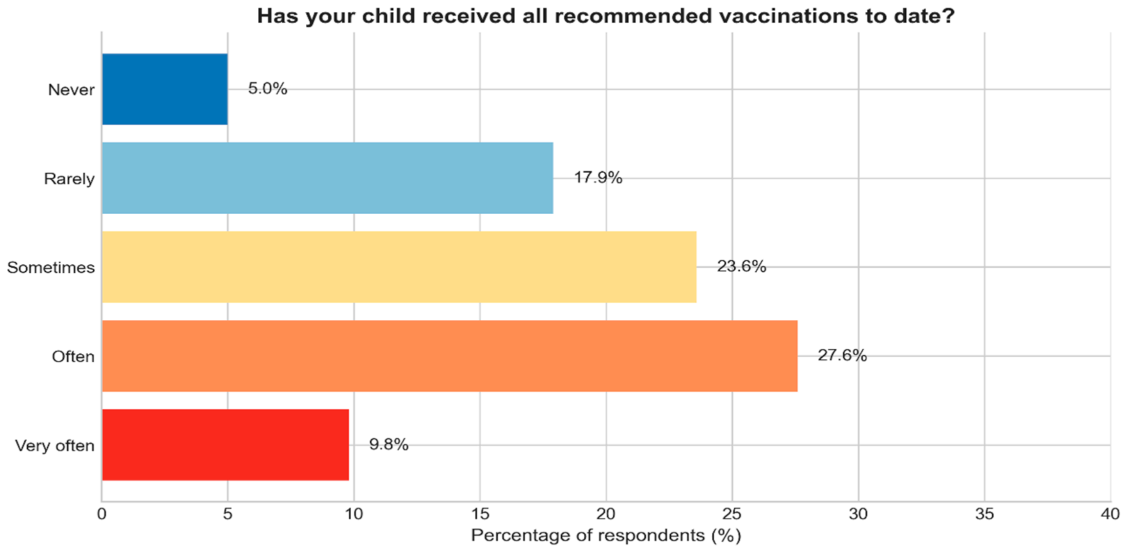

14. Is your child up to date on all recommended vaccinations?

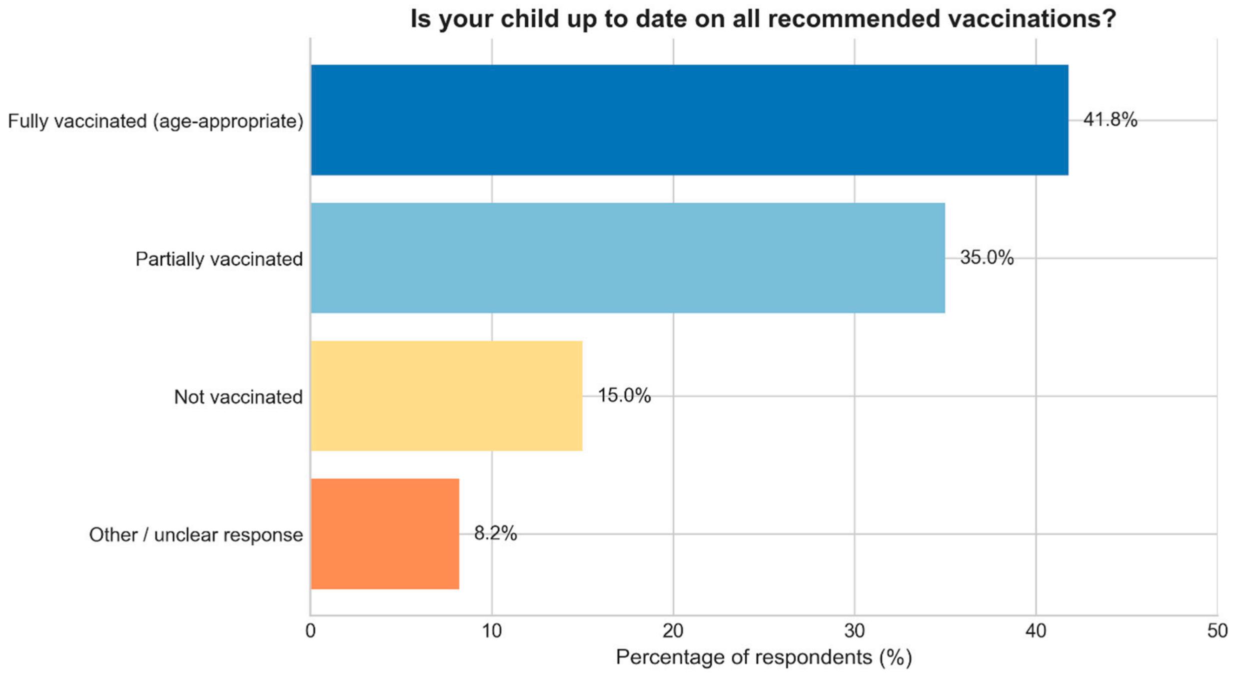

15. Which vaccinations were missed or delayed?

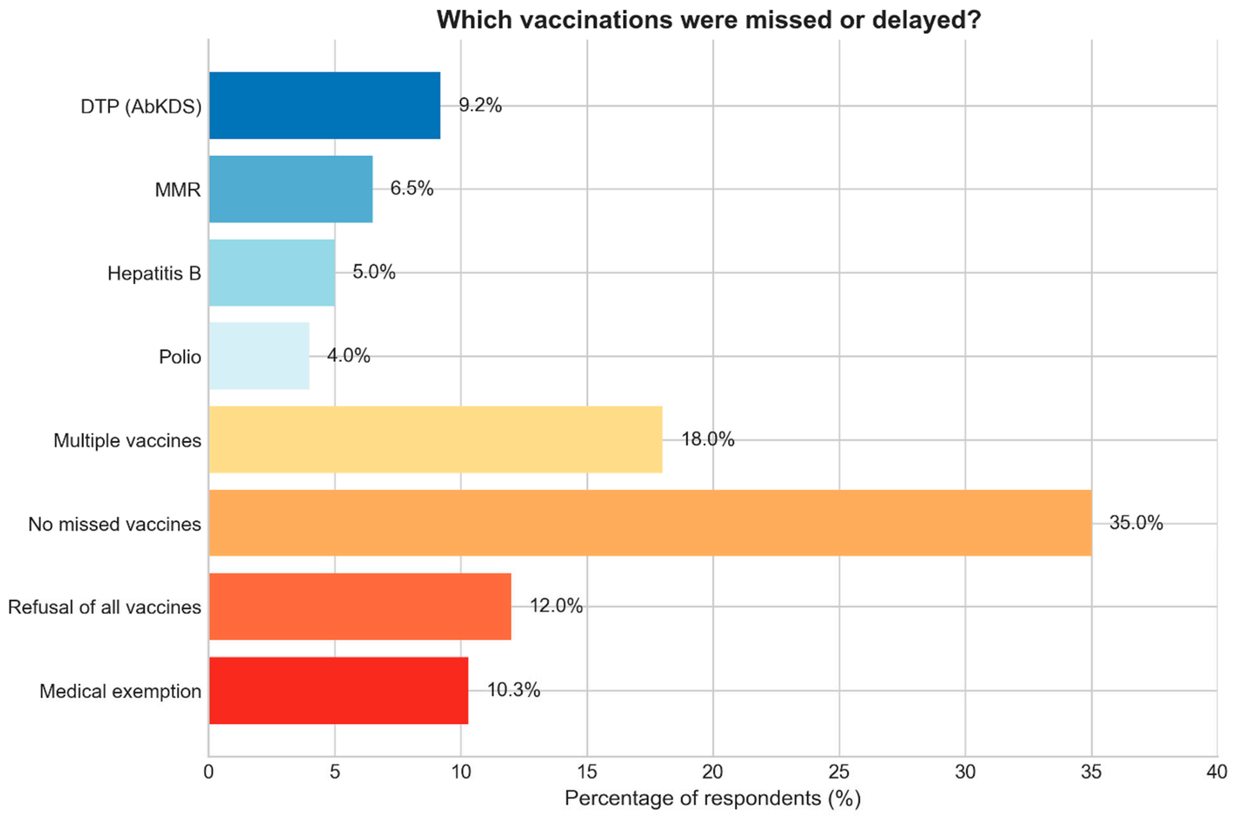

16. If vaccinations were not received, what were the primary reasons?

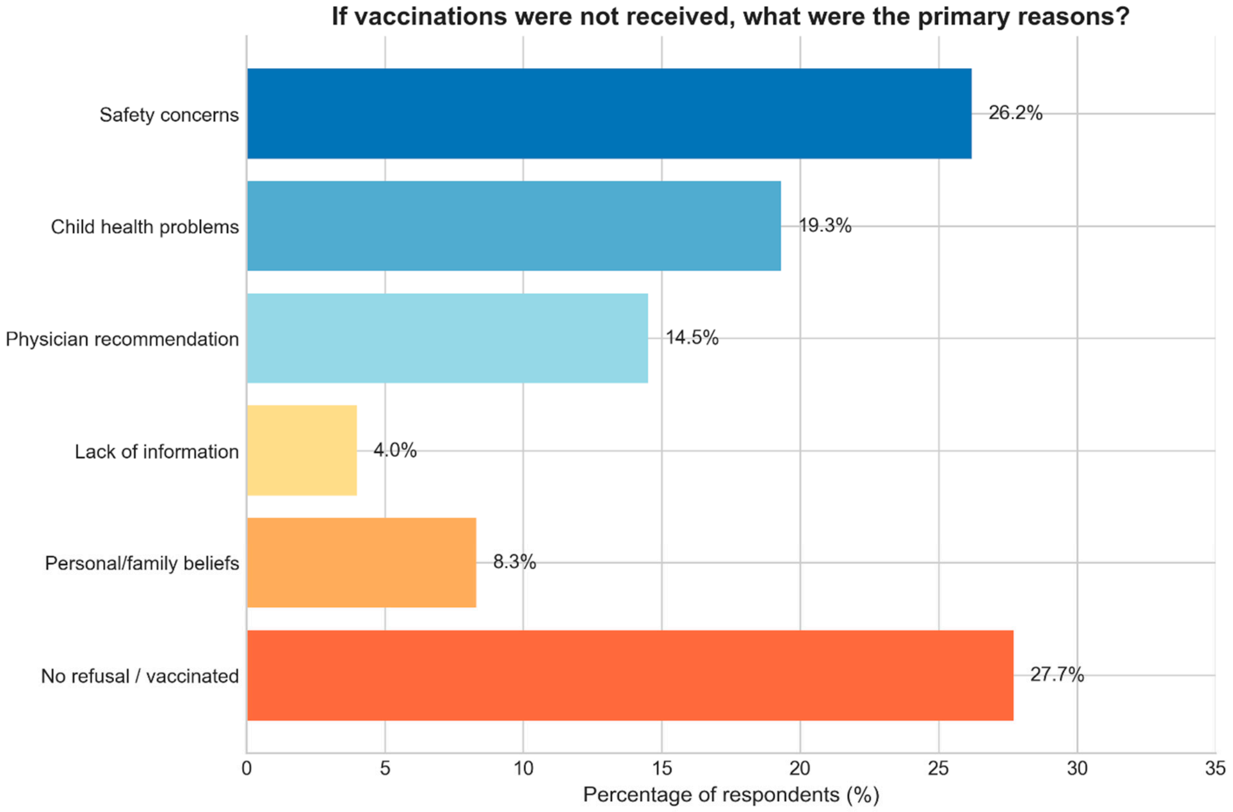

17. Pertussis remains a serious infection, especially for young children. How concerned are you about the possibility of your child catching it?

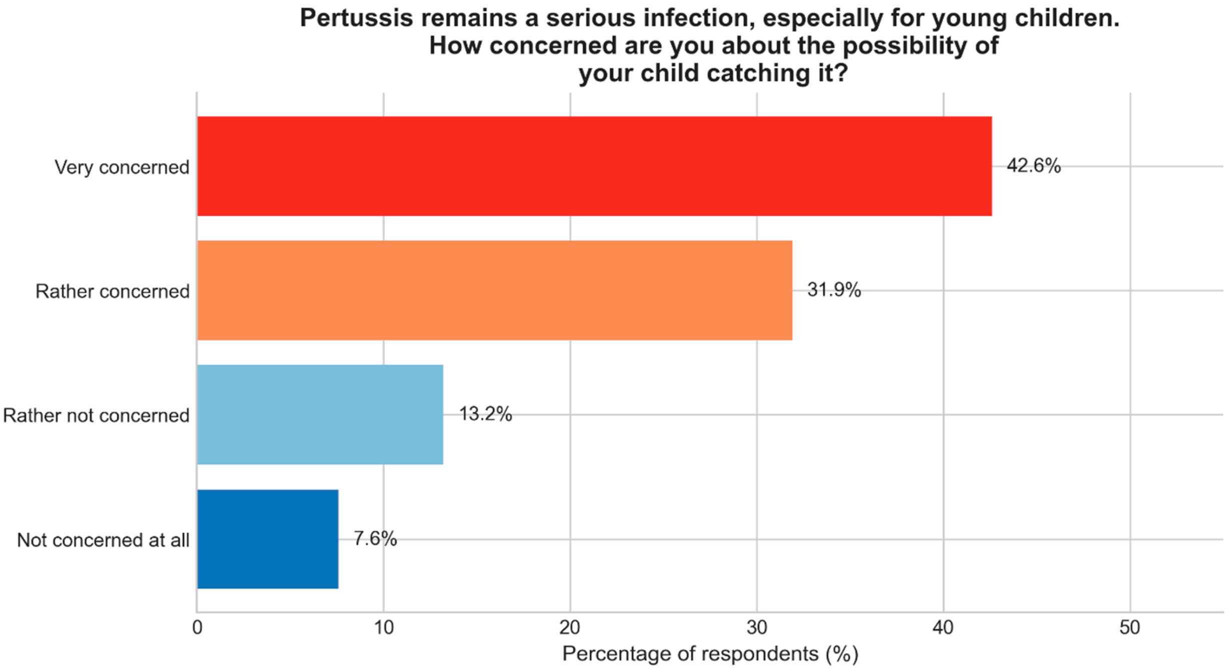

18. Measles remains one of the most contagious infections and can lead to serious complications, especially in young children. How concerned are you about the risk of your child becoming infected?

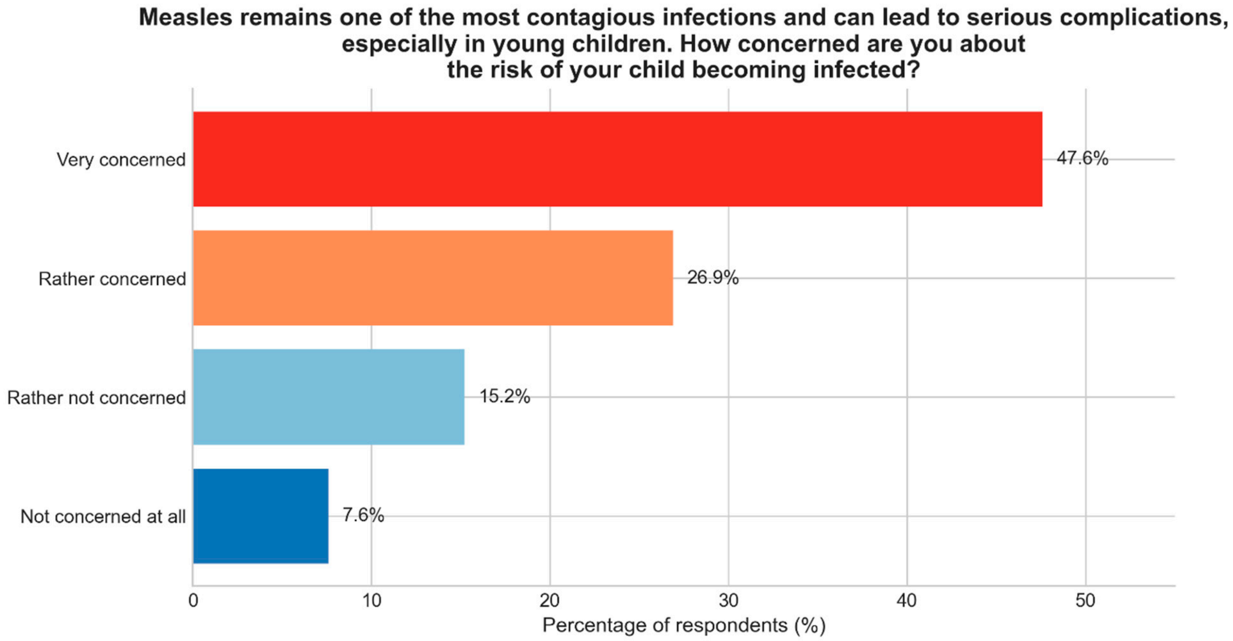

19. Do you believe that follow-up (catamnestic) centers should provide vaccination consultations for children with special medical needs, while leaving the actual administration of vaccines to local primary care clinics?

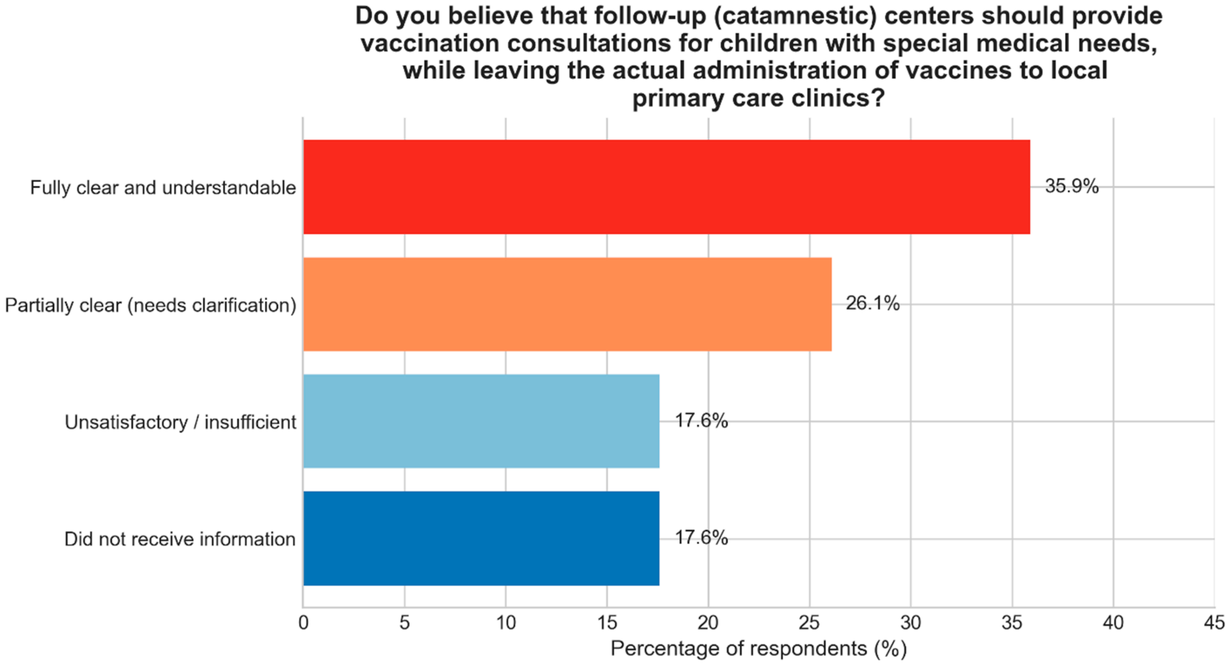

20. What information about vaccination were you given by healthcare professionals?

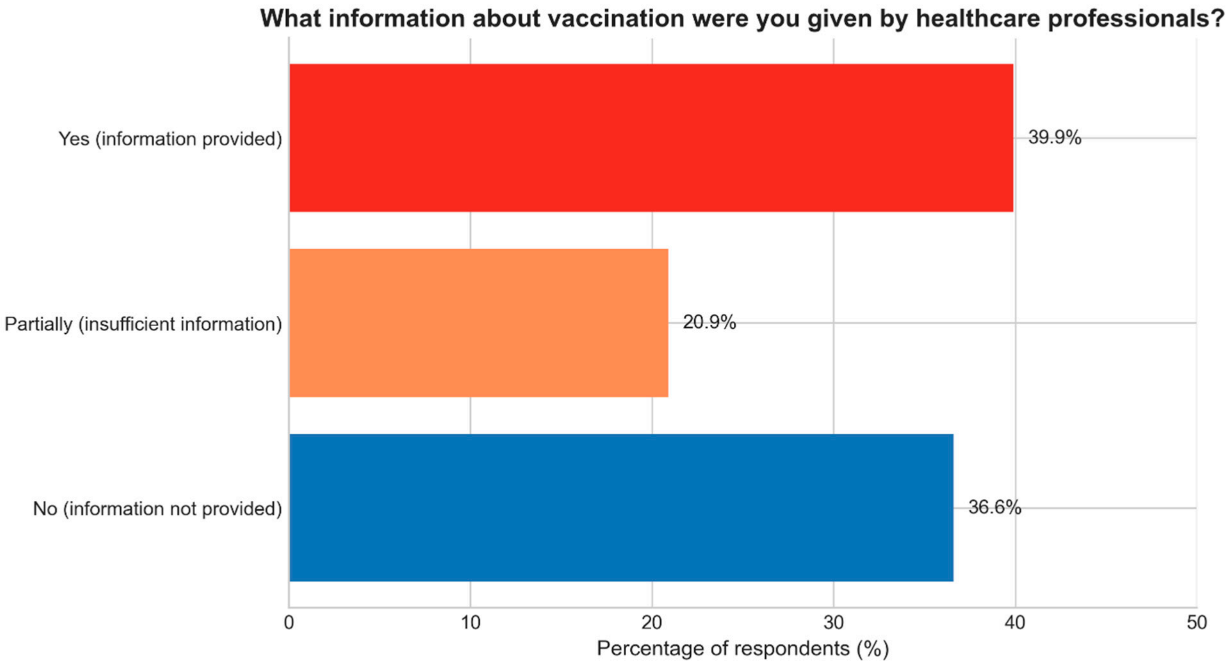

21. Have you received consultations from specialists (such as a neurologist or immunologist) regarding your child's vaccination?

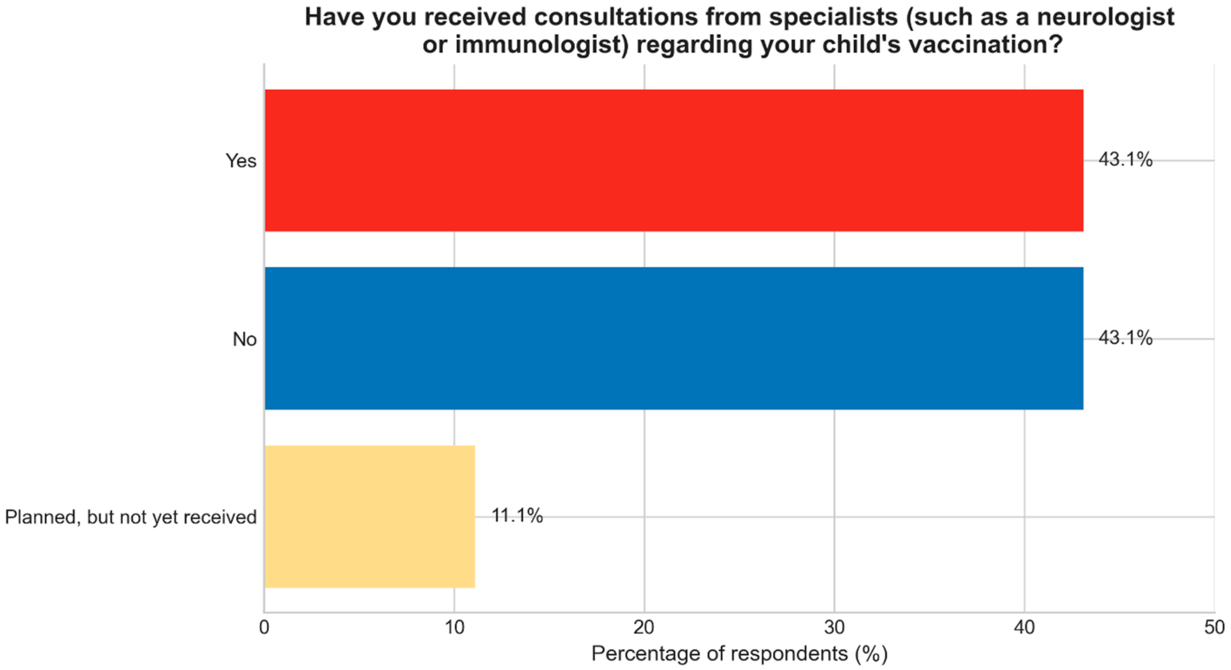

22. What reactions did you observe in your child after vaccinations (if any were given)?

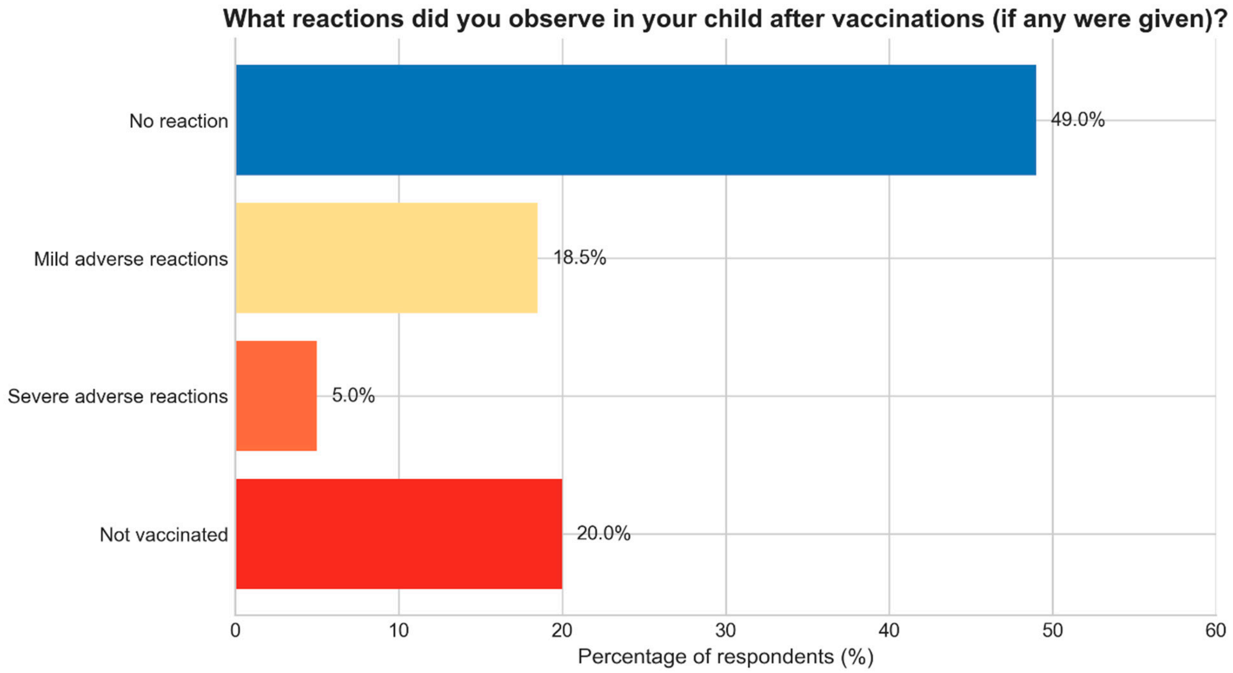

23. In your opinion, what measures could help you better understand and make a decision about your child’s vaccination?

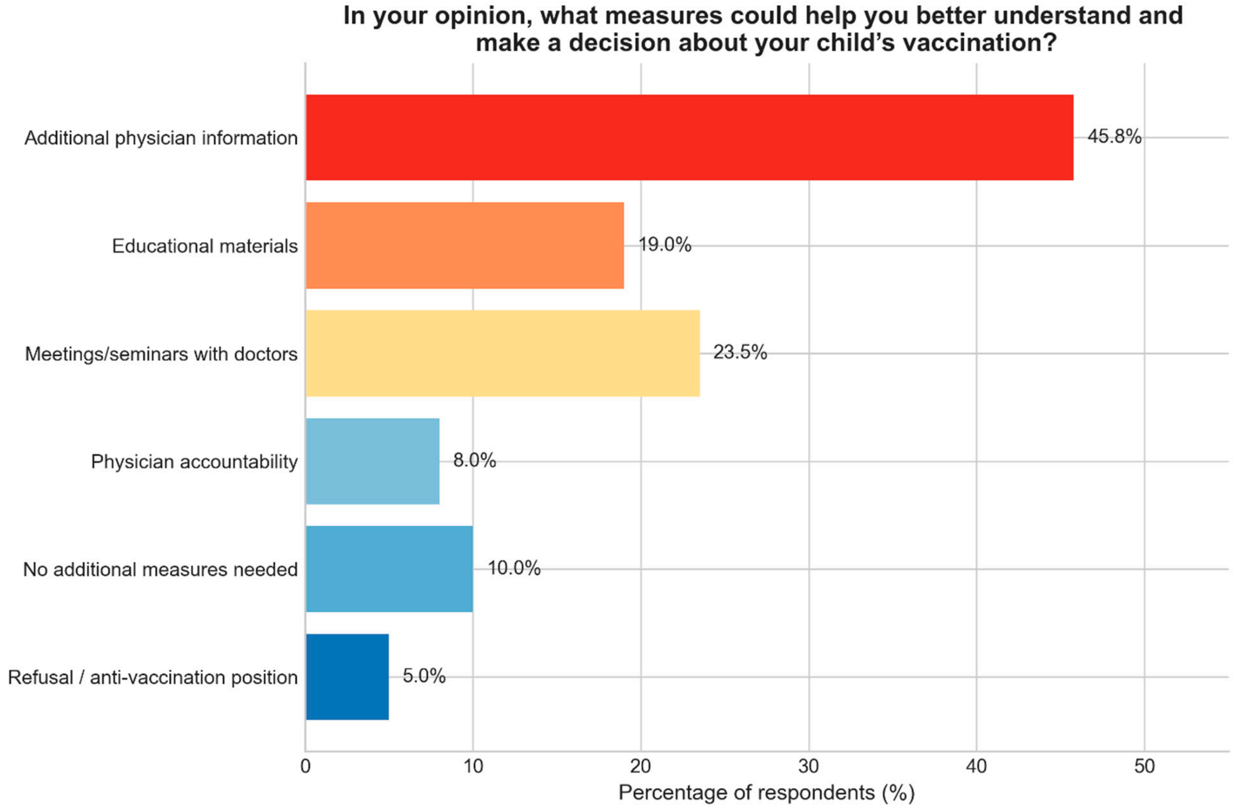

24. How would you rate the overall accessibility and quality of medical information regarding vaccination for parents of preterm infants?

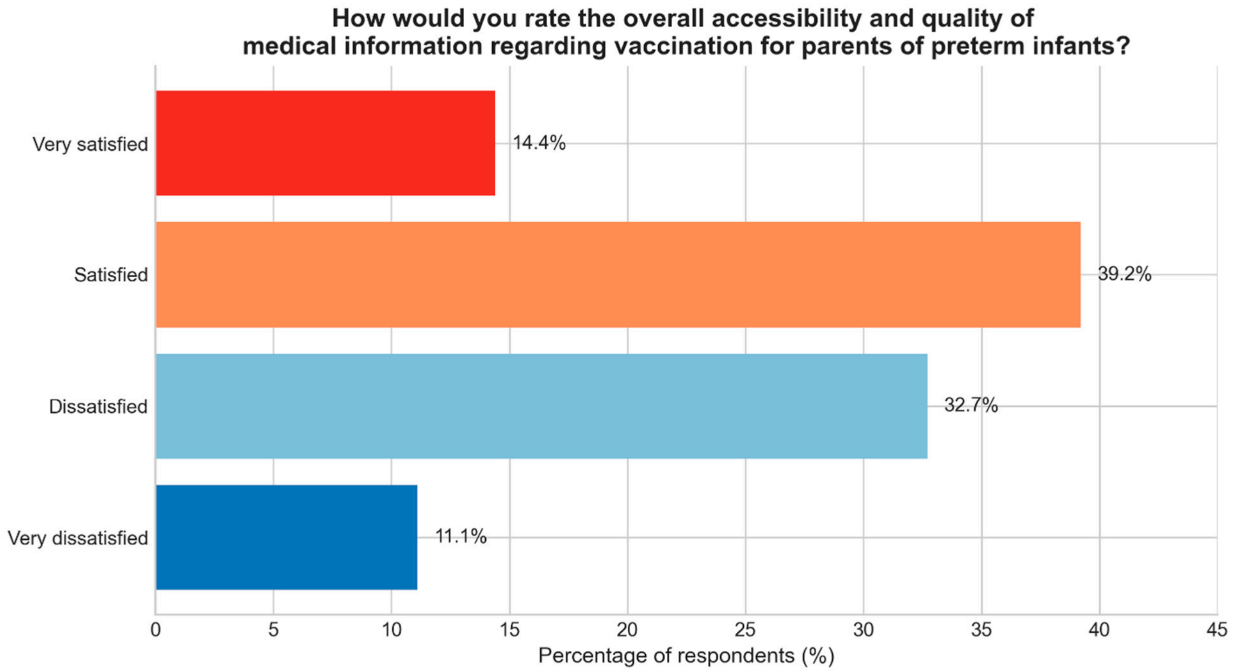

III. Managers Survey

Reliability and Internal Consistency of the Managers' Survey

To evaluate the psychometric reliability of the questionnaire administered to healthcare managers (N = 43), an internal consistency analysis was conducted. The instrument demonstrated robust reliability, with an overall Cronbach's alpha coefficient of $\alpha = 0.869 (95%CI [0.803, 0.920]). This value substantially exceeds the conventionally accepted methodological threshold of 0.70, indicating excellent internal consistency and demonstrating that the survey variables reliably capture cohesive structural and organizational perspectives regarding vaccination management.

Factor	Meaning
Factor1	accessibility + communication + coordination
Factor2	organizational support + reminder systems
Factor3	public awareness + information barriers

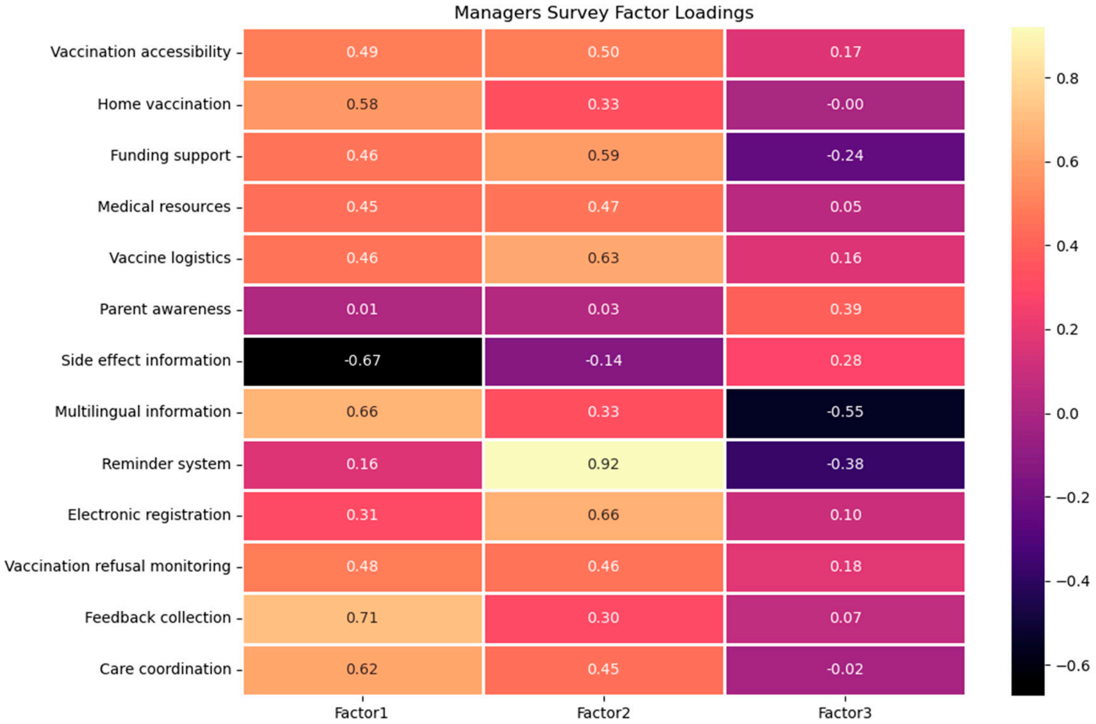

25. The vaccination clinic schedule in the region is adapted to the population (including evening and weekend hours).

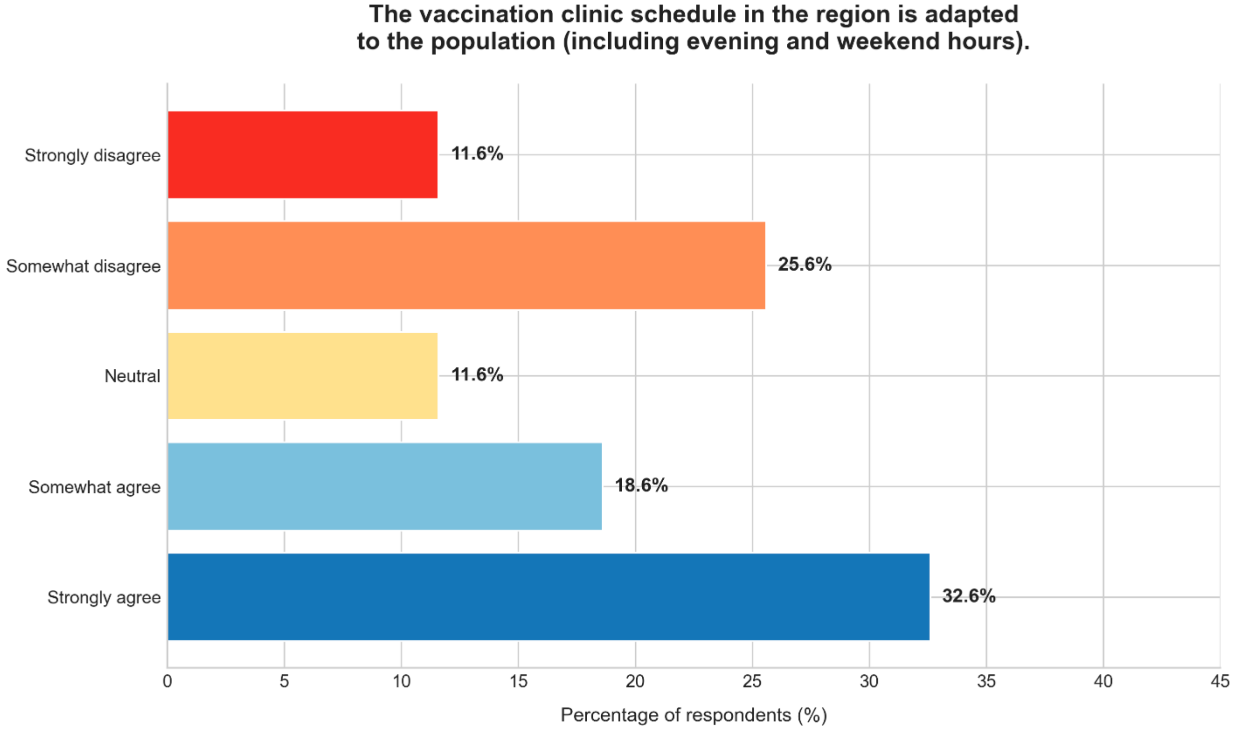

26. Does your region provide home-based vaccination for children with limited mobility or for families living in hard-to-reach areas (for example, through mobile vaccination teams)?

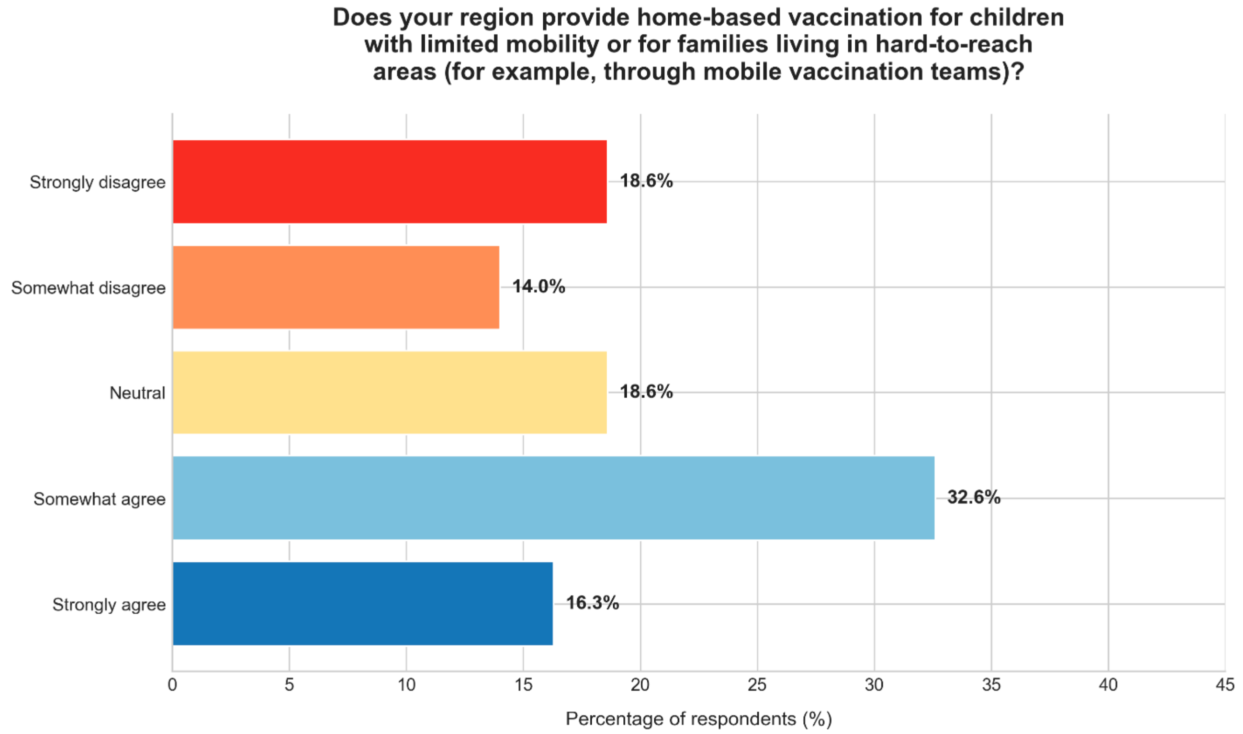

27. All patients, including children, receive vaccines free of charge under the national immunization schedule.

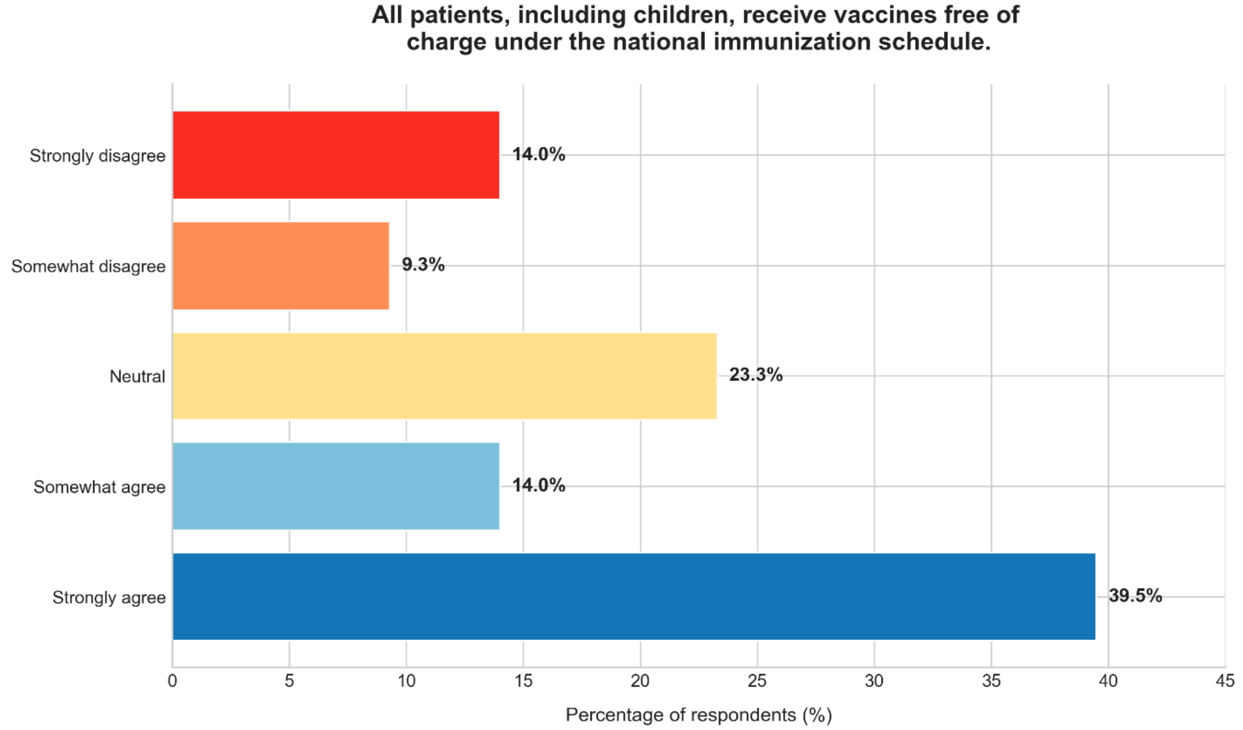

28. Additional funding for personnel training, logistics, and public nawareness campaigns is allocated in an adequate amount. CDC Public

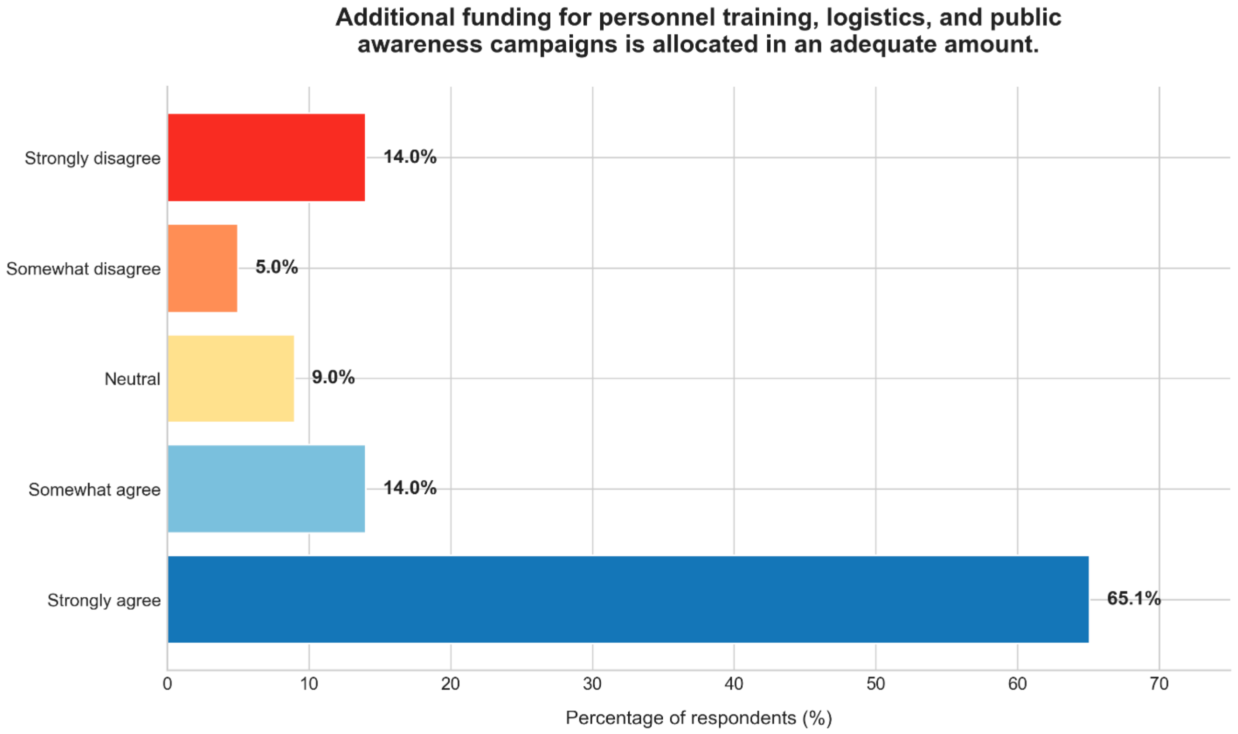

29. Have there been any vaccine supply delays in your region over the past year?

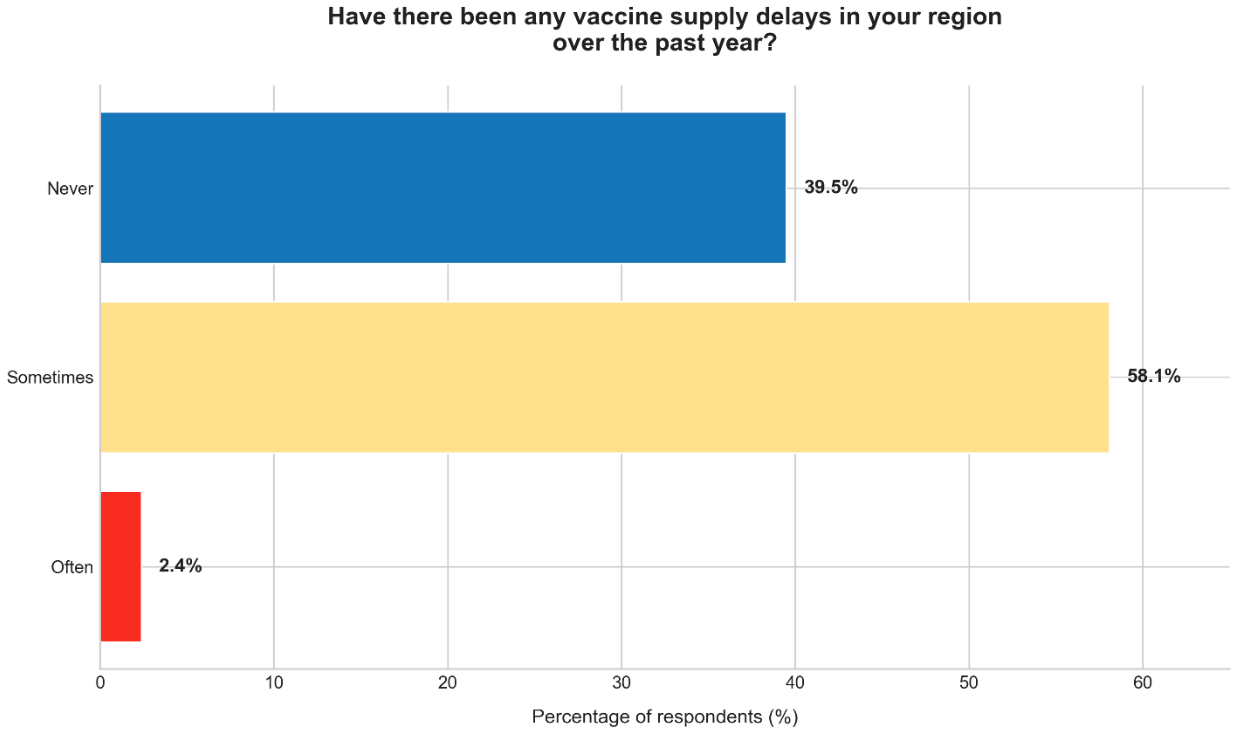

30. Public awareness campaigns regarding the importance of vaccination are regularly conducted in the region.

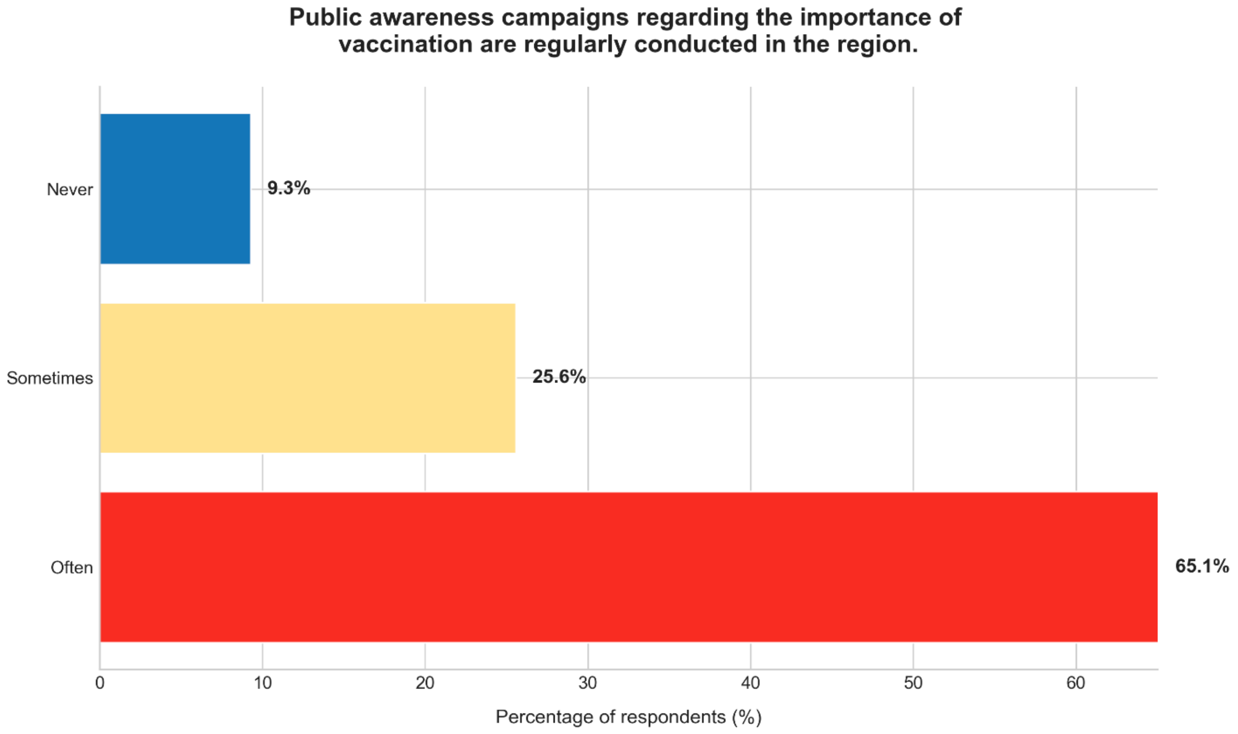

31. Parents receive transparent and reliable information about the side effects of vaccines available in Kazakhstan.

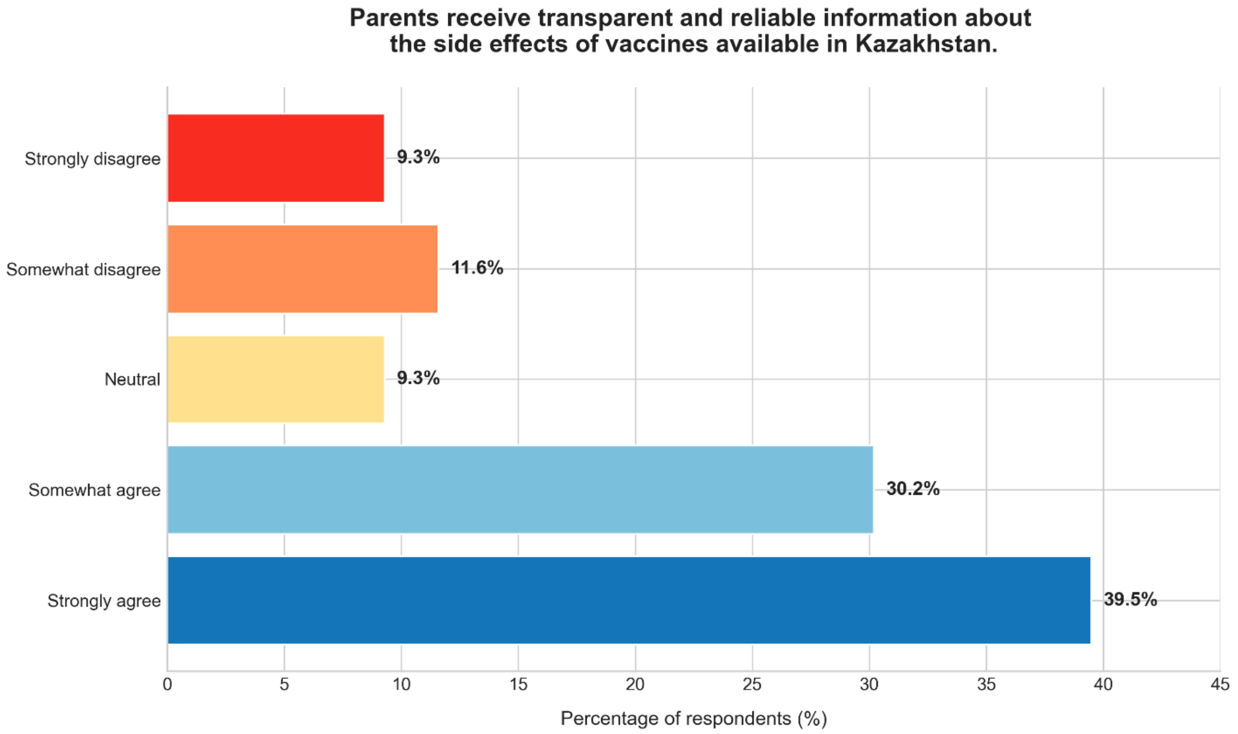

32. Vaccination information is provided in accessible languages (Kazakh, Russian, etc.)

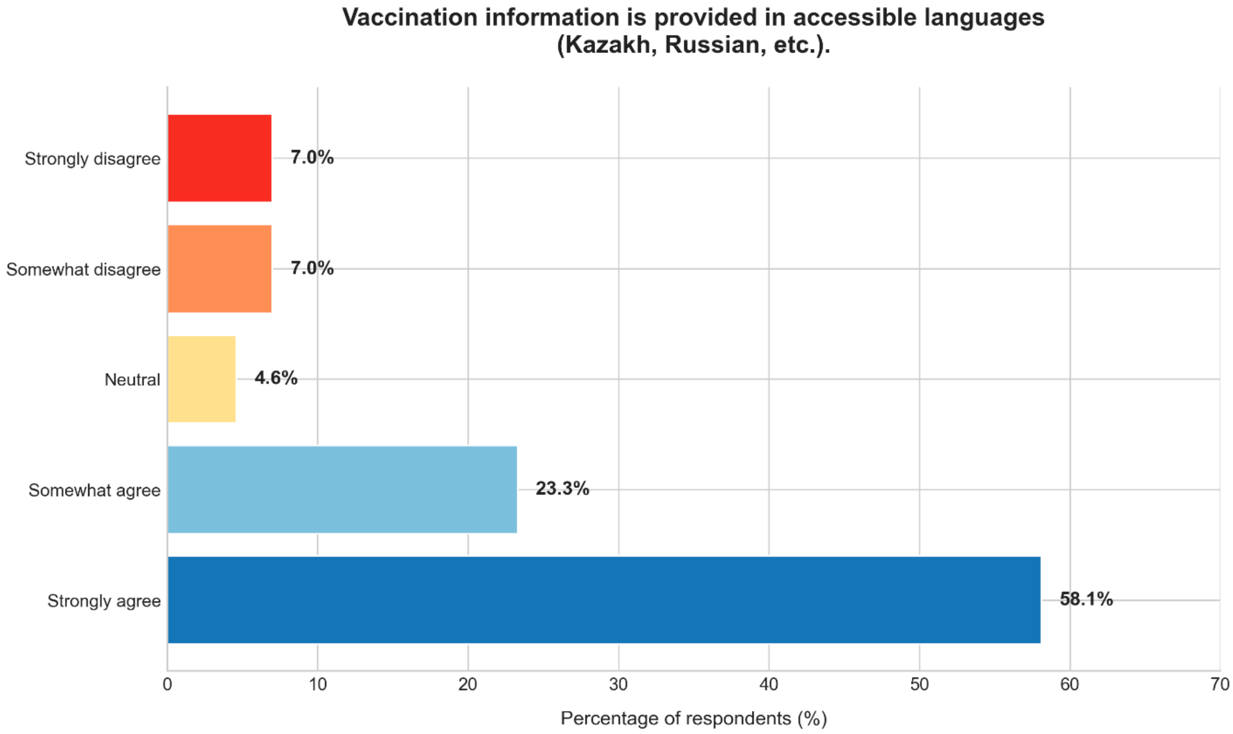

33. Parents regularly receive vaccination reminders (e.g., via SMS, phone calls, or mobile applications).

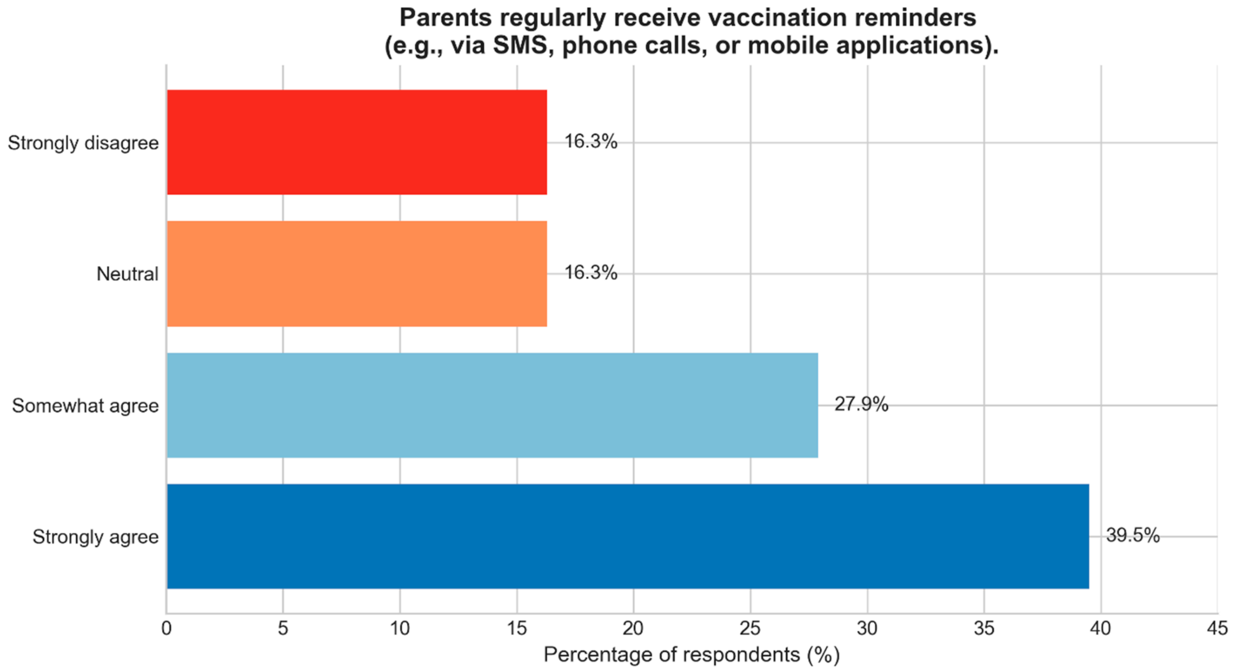

34. Does your organization use an electronic system for vaccine registration and tracking (e.g., within a Medical Information System)?

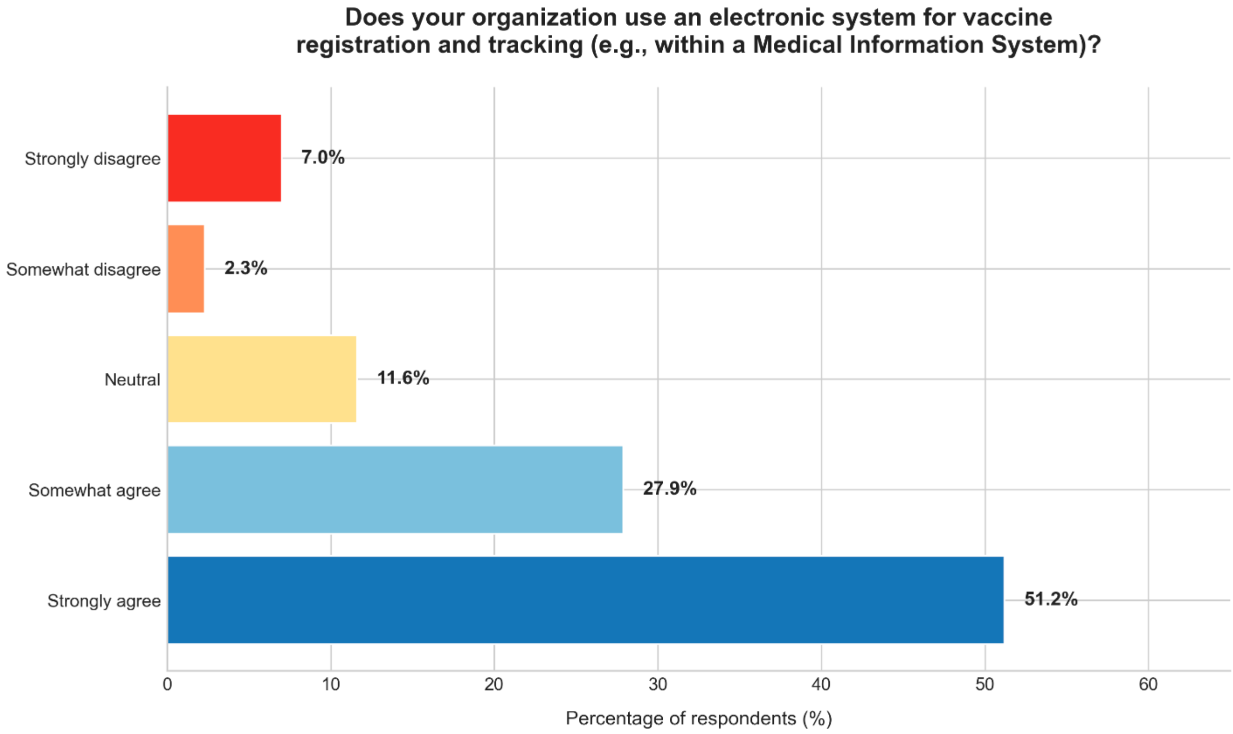

35. Does your organization use an electronic system for vaccine registration and tracking (e.g., within a Medical Information System)?

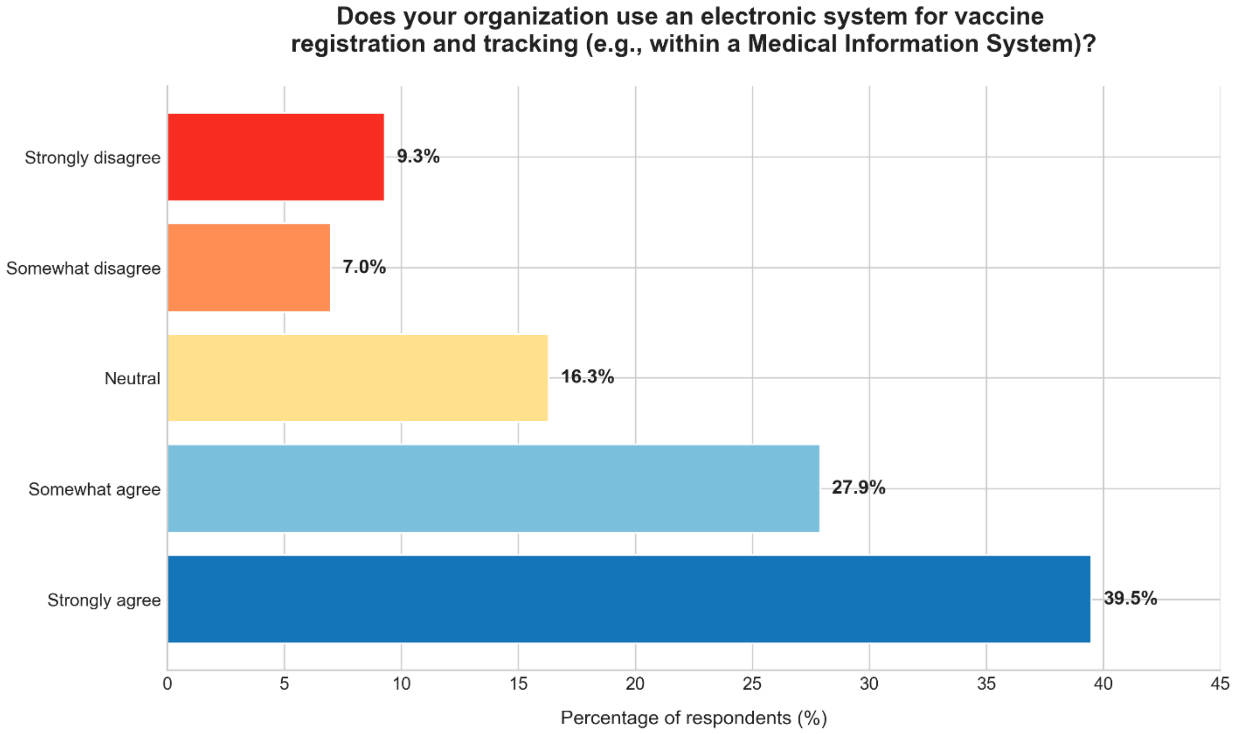

36. Does your facility maintain a feedback loop with parents following vaccination through tools such as surveys, questionnaires, or a dedicated hotline?

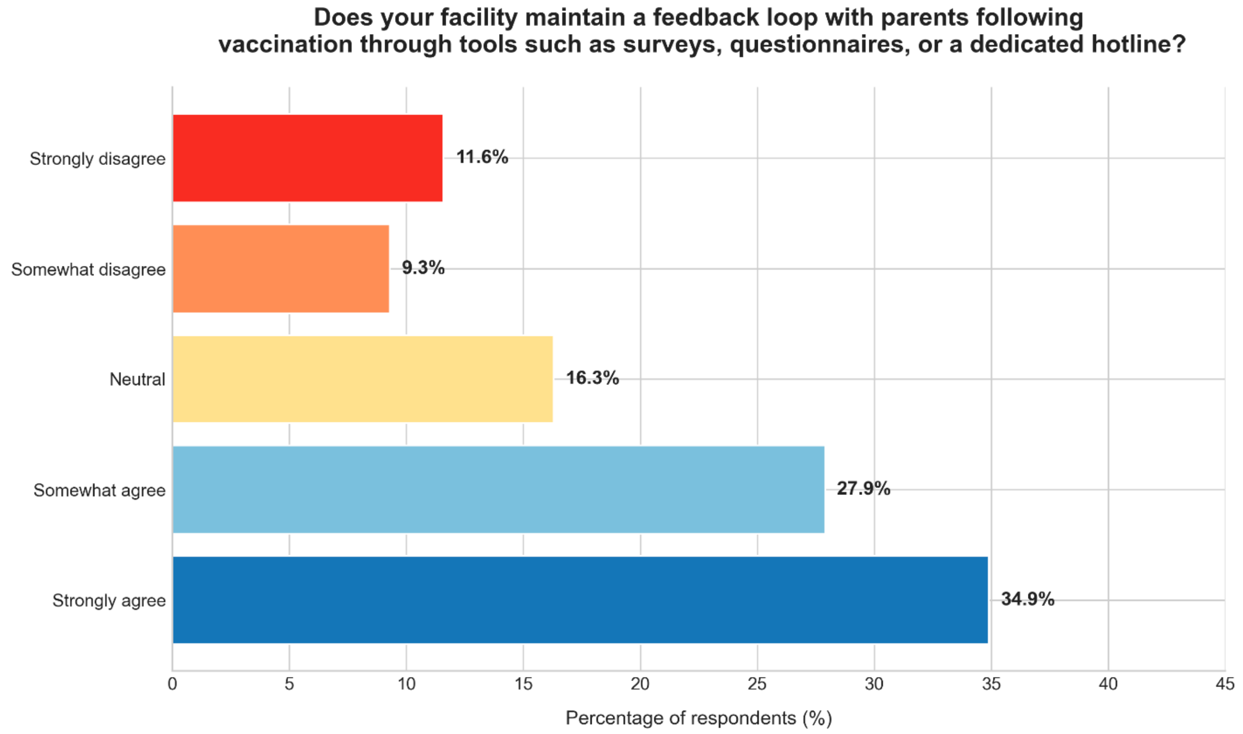
Does your facility maintain a feedback loop with parents following vaccination through tools such as surveys, questionnaires, or a dedicated hotline?
Strongly disagree
11.6%
Somewhat disagree
9.3%
Neutral
16.3%
Somewhat agree
27.9%
Strongly agree
34.9%
0
5
10
15
20
25
30
35
40
45
Percentage of respondents (%)
