# Supplementary figures and images for: Immunization Gaps Among High-Risk Preterm Infants in Kazakhstan
Source: Vaccines (Basel). 2026 Jul 20;14(7):638. doi: 10.3390/vaccines14070638 (PMC13431342; doi:10.3390/vaccines14070638)

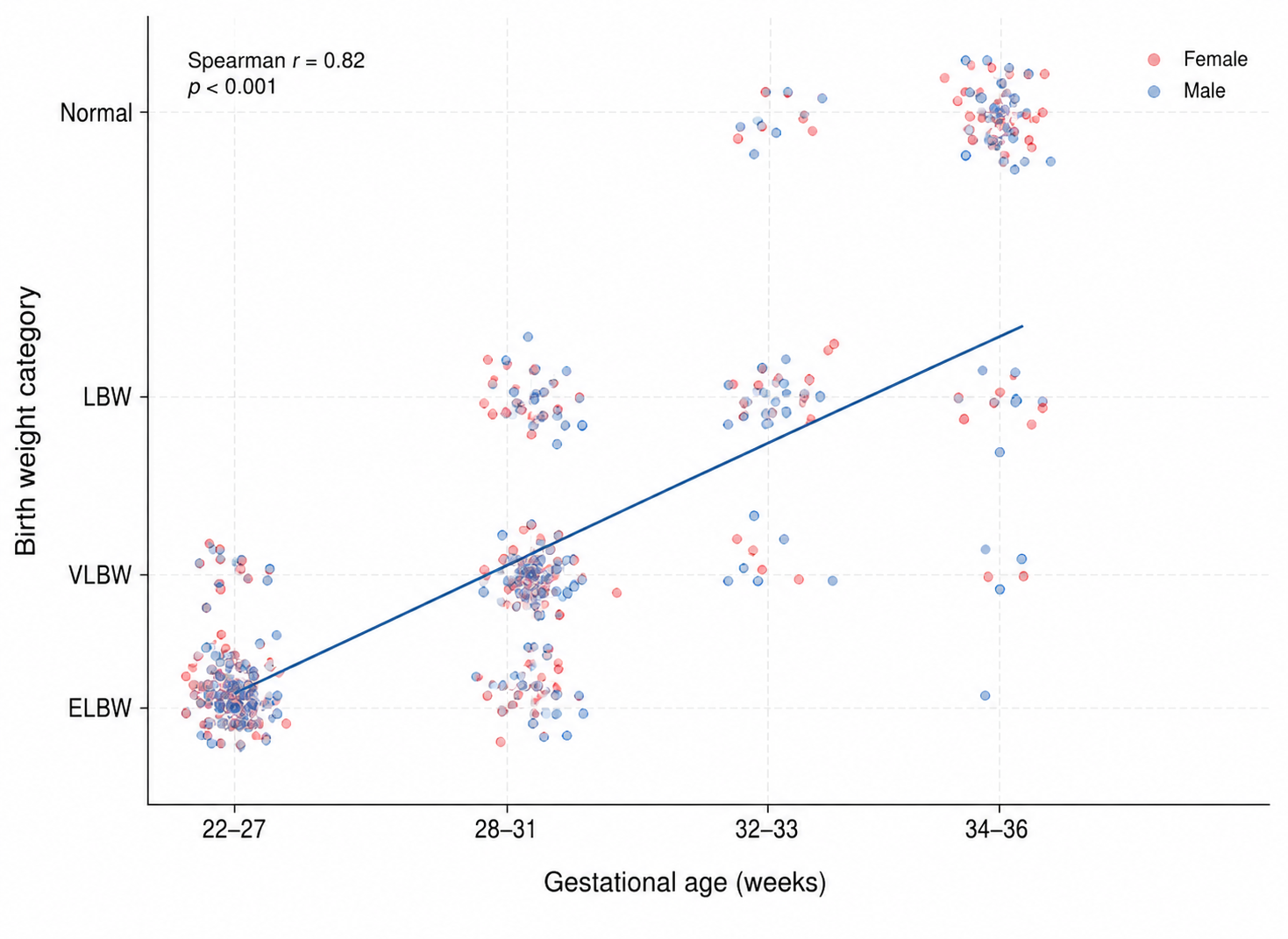

Supplement: Supplementary file 1 [file vaccines-14-00638-s001.zip › Supplementary Figure S1 Association between gestational age and birth weight categories in preterm infant.png]

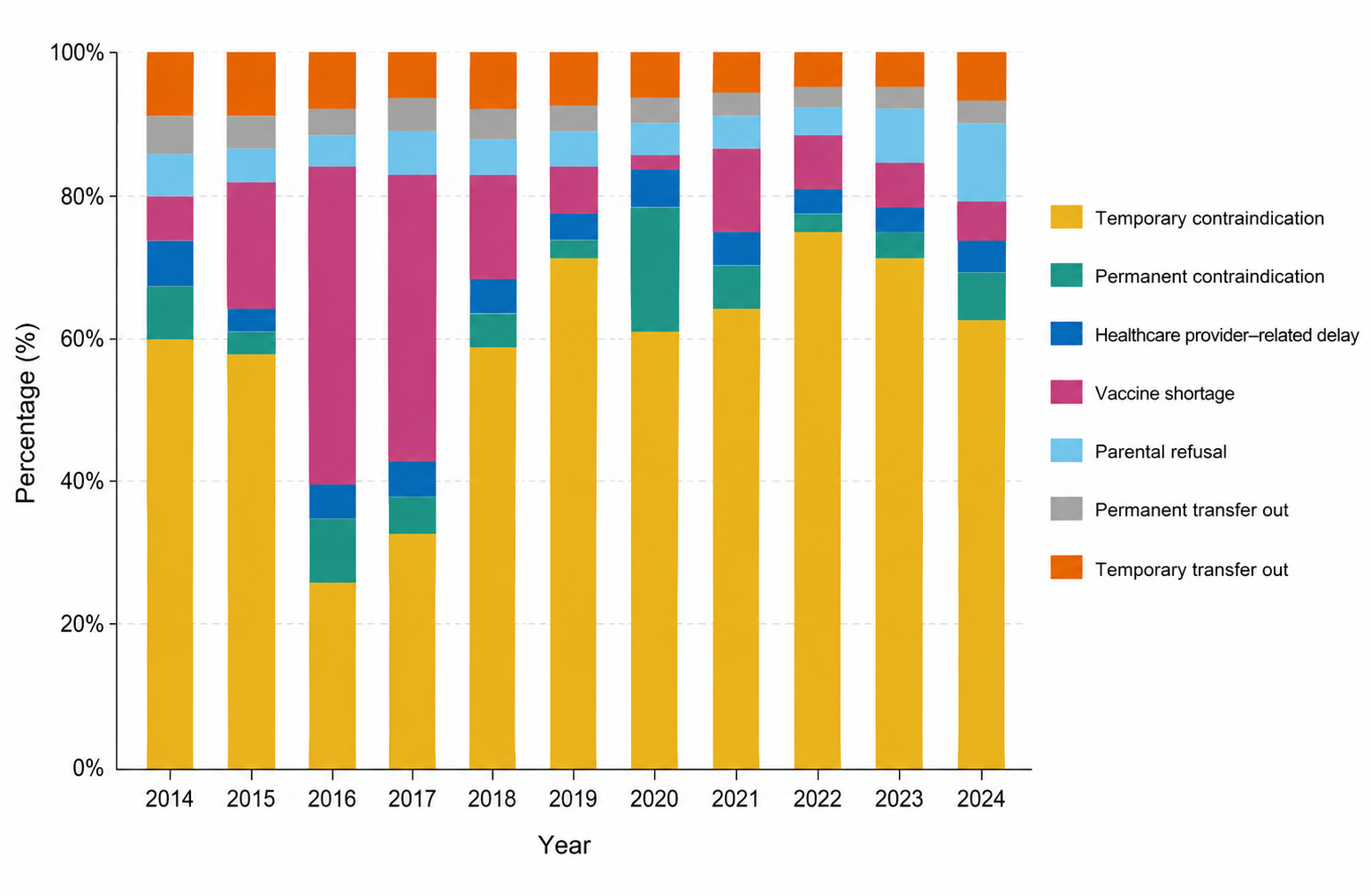

Supplement: Supplementary file 1 [file vaccines-14-00638-s001.zip › Supplementary Figure S2. Distribution of reported reasons for delayed DPT vaccination in Kazakhstan from 2014 to 2024..png]

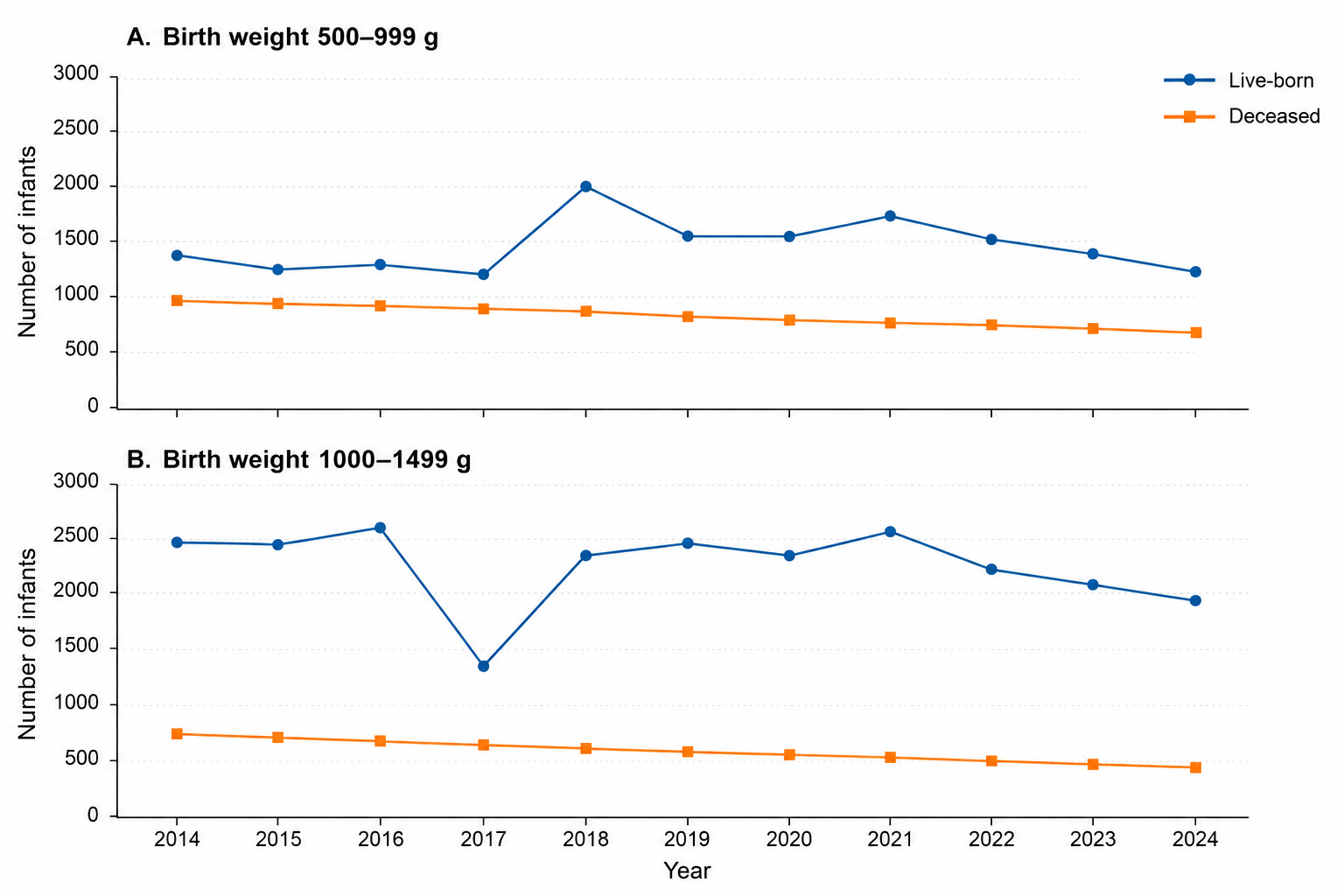

Supplement: Supplementary file 1 [file vaccines-14-00638-s001.zip › Supplementary Figure S3.Live-born and deceased infants by birth weight categorie.png]

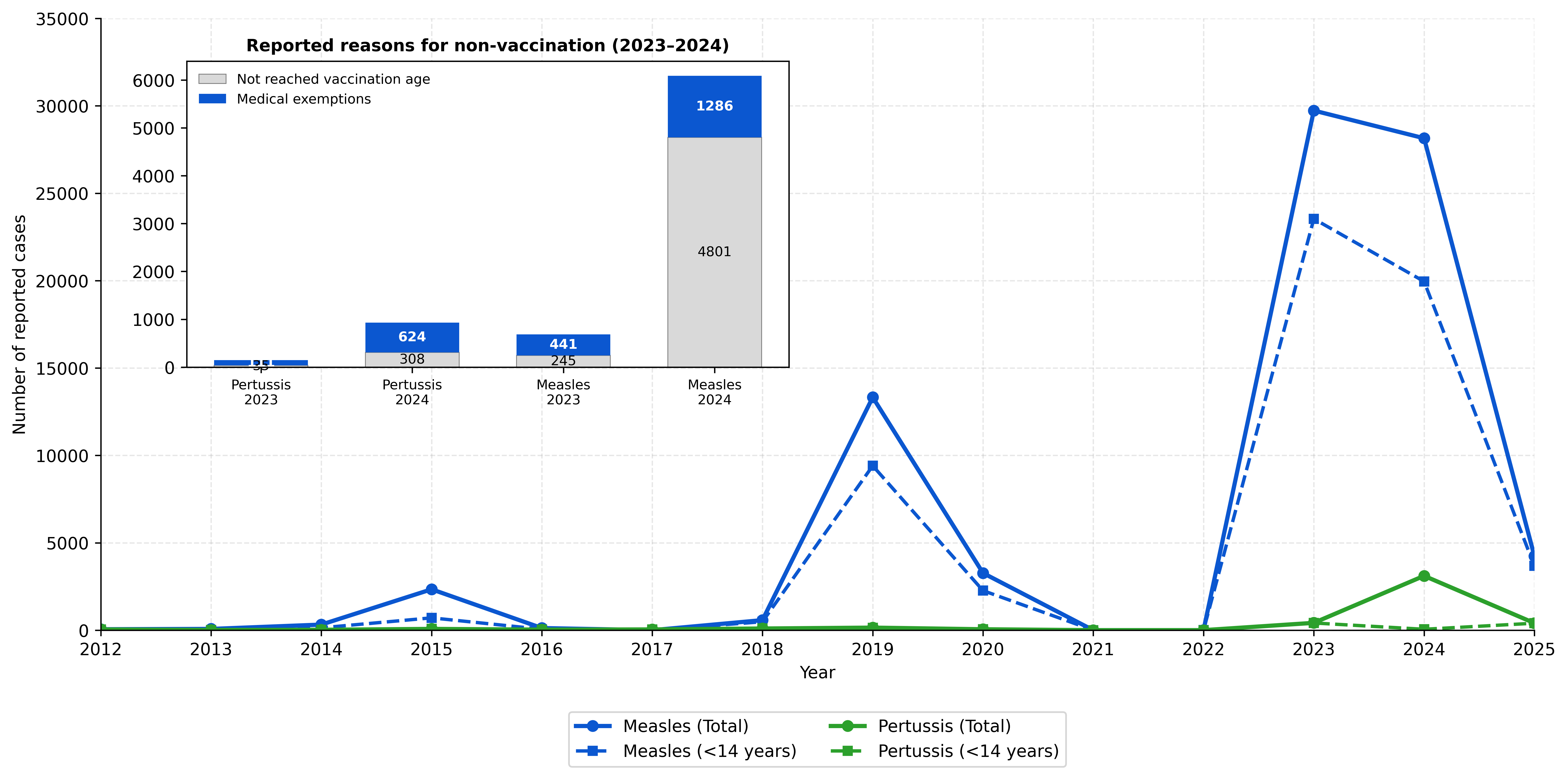

Supplement: Supplementary file 1 [file vaccines-14-00638-s001.zip › Supplementary Figure S4. National trends in reported measles and pertussis cases and reported reasons for non-vaccination in Kazakhstan, 2012–2025.png]

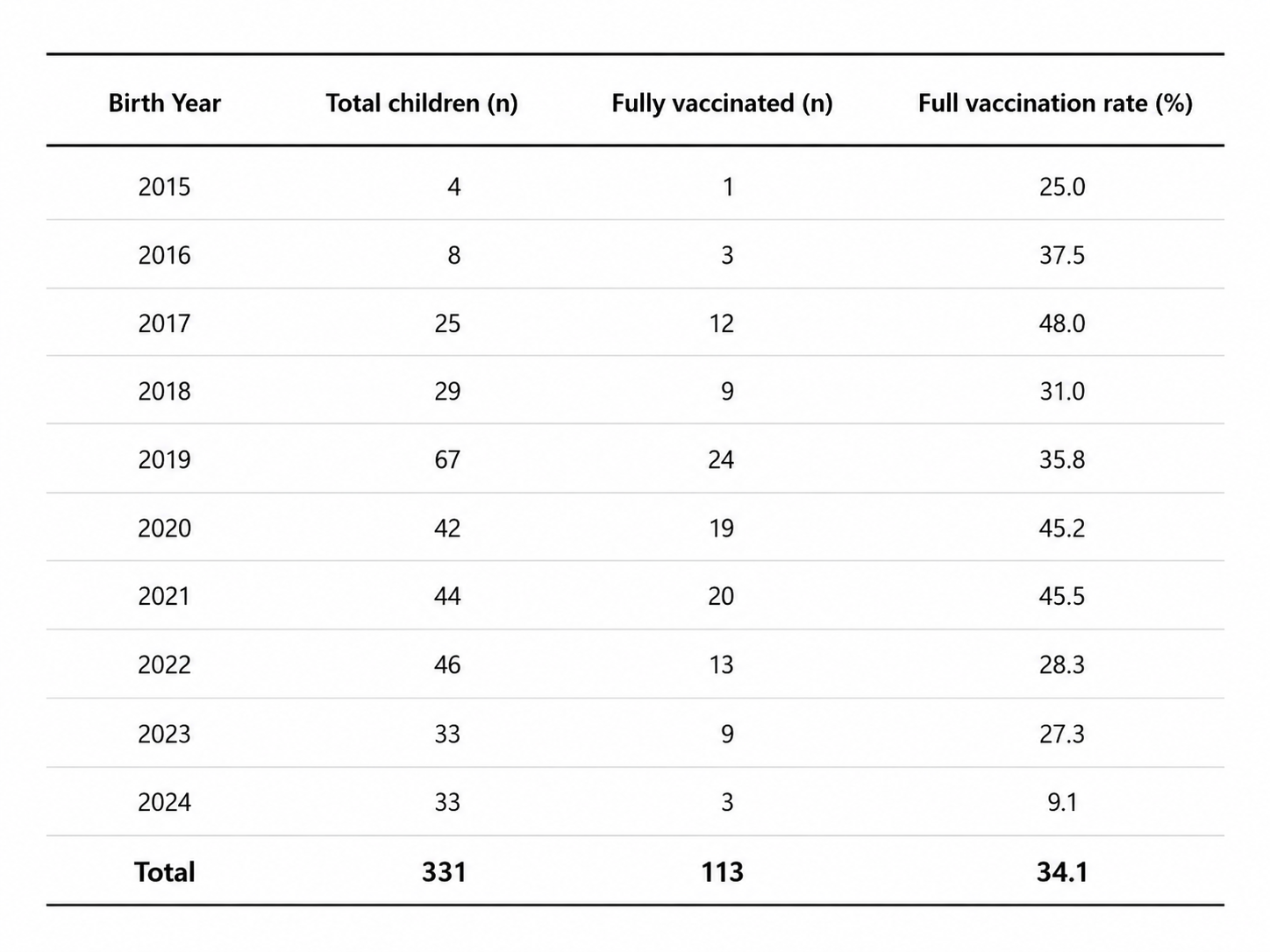

Supplement: Supplementary file 1 [file vaccines-14-00638-s001.zip › Supplementary Table S1. Temporal trends in full vaccination coverage by birth year among high-risk preterm infants (2015–2024) (1).png]

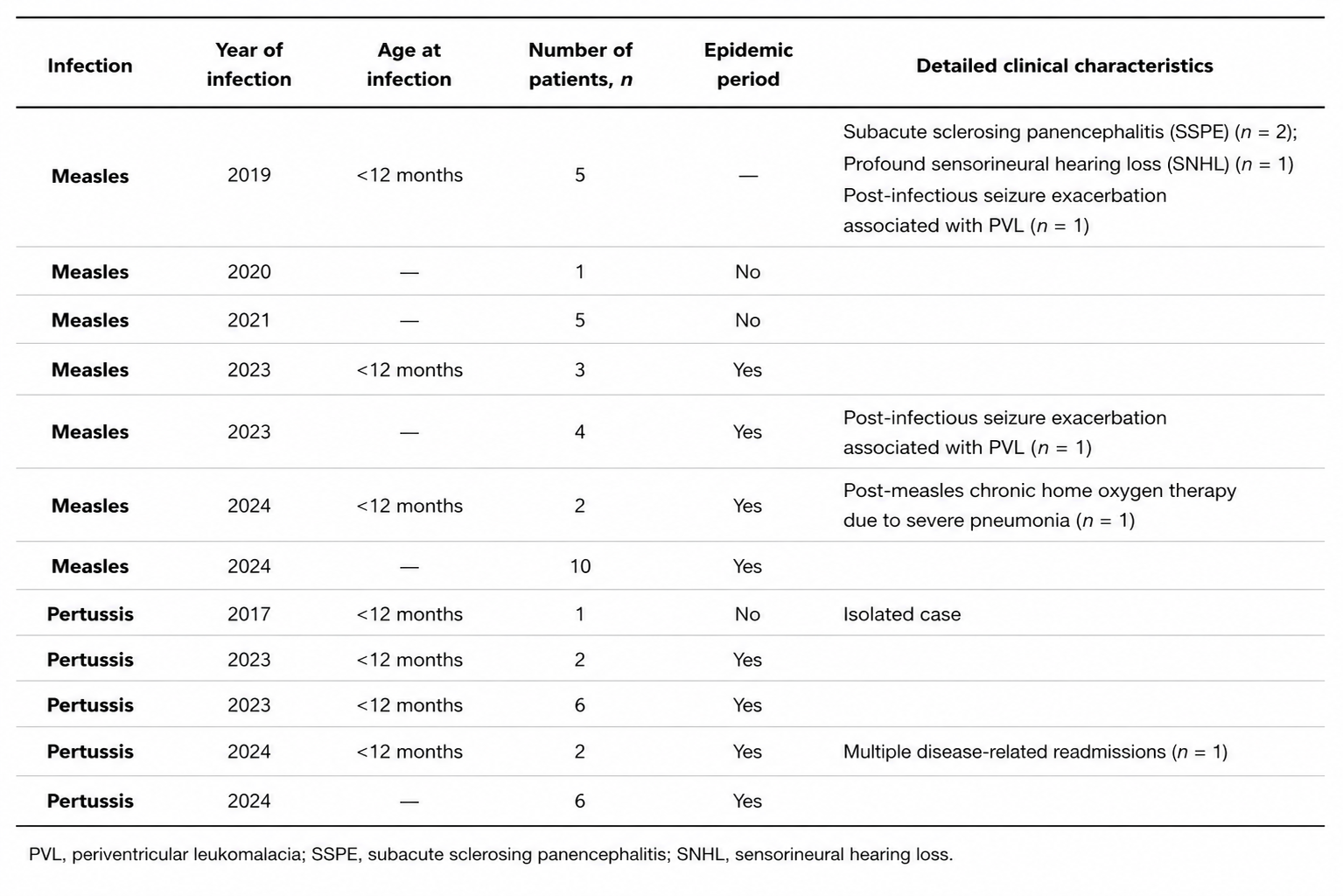

Supplement: Supplementary file 1 [file vaccines-14-00638-s001.zip › Supplementary Table S2. Detailed clinical characteristics of vaccine-preventable infections in preterm infants.png]

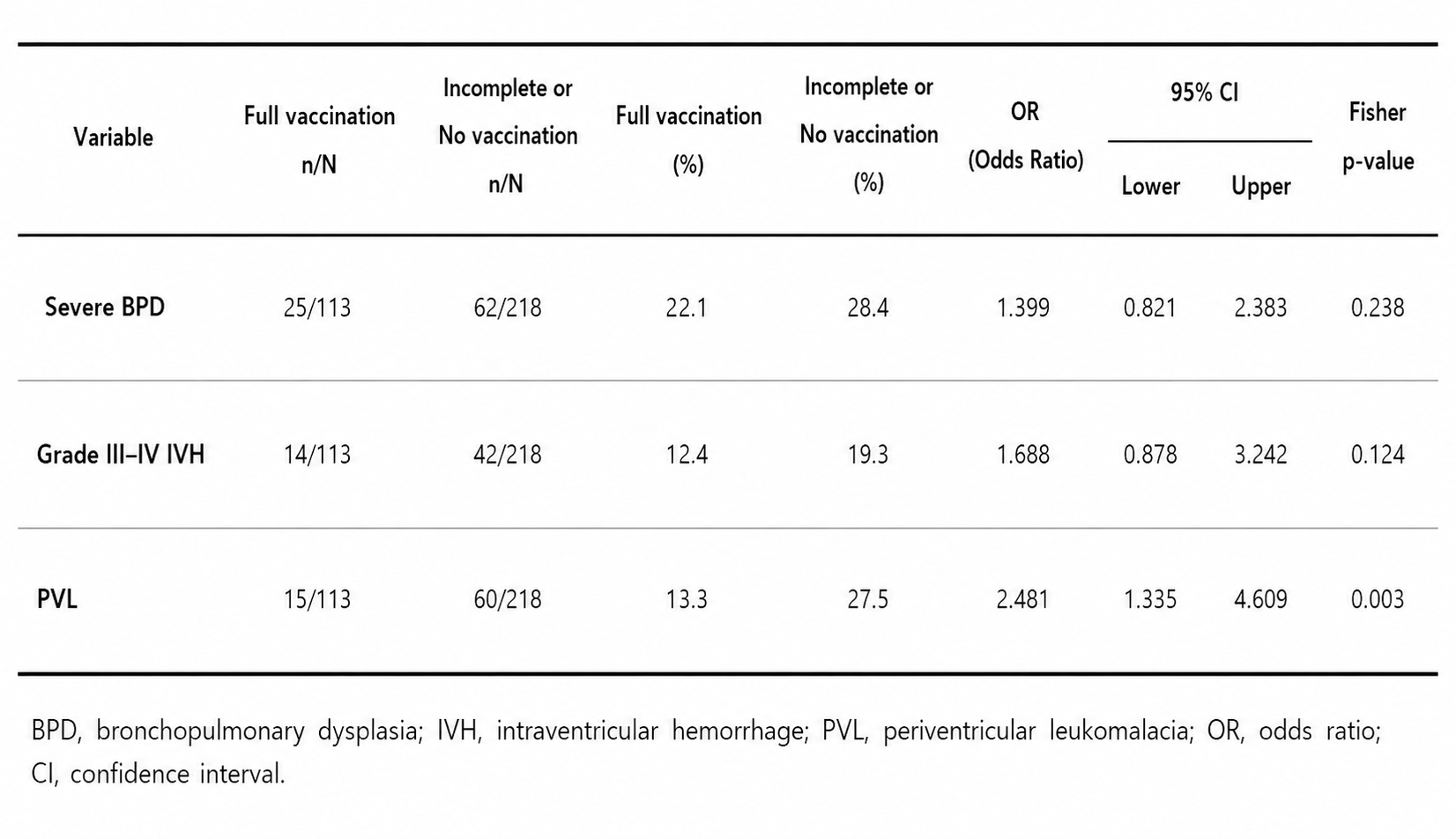

Supplement: Supplementary file 1 [file vaccines-14-00638-s001.zip › Supplementary Table S3. Univariate analysis of selected clinical risk factors associated with incomplete or no vaccination in preterm infants by 24 months of age.png]
